# Rewiring host–microbe interactions and barrier function during gastrointestinal inflammation

**DOI:** 10.1093/gastro/goac008

**Published:** 2022-03-11

**Authors:** Sune K Jensen, Simone I Pærregaard, Emma P Brandum, Astrid S Jørgensen, Gertrud M Hjortø, Benjamin A H Jensen

**Affiliations:** Department of Biomedical Sciences, Faculty of Health and Medical Sciences, University of Copenhagen, Copenhagen, Denmark

**Keywords:** host-defense peptides, mucosal barrier defects, IBD, nutritional immunology, bacterial translocation

## Abstract

Organismal survival depends on a well-balanced immune system and maintenance of host–microbe mutualism. The fine-tuned relationship between the gut microbiota and host immunity is constantly challenged by opportunistic bacteria testing the integrity of gastrointestinal (GI) barrier defenses. Barrier dysfunction reduces immunological tolerance towards otherwise innocuous microbes; it is a process that may instigate chronic inflammation. Paradoxically, sustained inflammation further diminishes barrier function, enabling bacterial translocation to extra-intestinal tissues. Once translocated, these bacteria stimulate systemic inflammation, thereby compromising organ function. While genetic risk alleles associate with barrier dysfunction, environmental stressors are key triggers of GI inflammation and associated breakdown in immune tolerance towards resident gut microbes. As dietary components dictate substrate availability, they also orchestrate microbiota composition and function, including migratory and pro-inflammatory potential, thus holding the capacity to fuel both GI and extra-intestinal inflammation. Additionally, Western diet consumption may weaken barrier defenses via curbed Paneth cell function and diminished host-defense peptide secretion. This review focuses on intervenable niches of host–microbe interactions and mucosal immunity with the ambition to provide a framework of plausible strategies to improve barrier function and regain tolerance in the inflamed mucosa via nutritional intervention.

## Introduction

**Figure 1.**
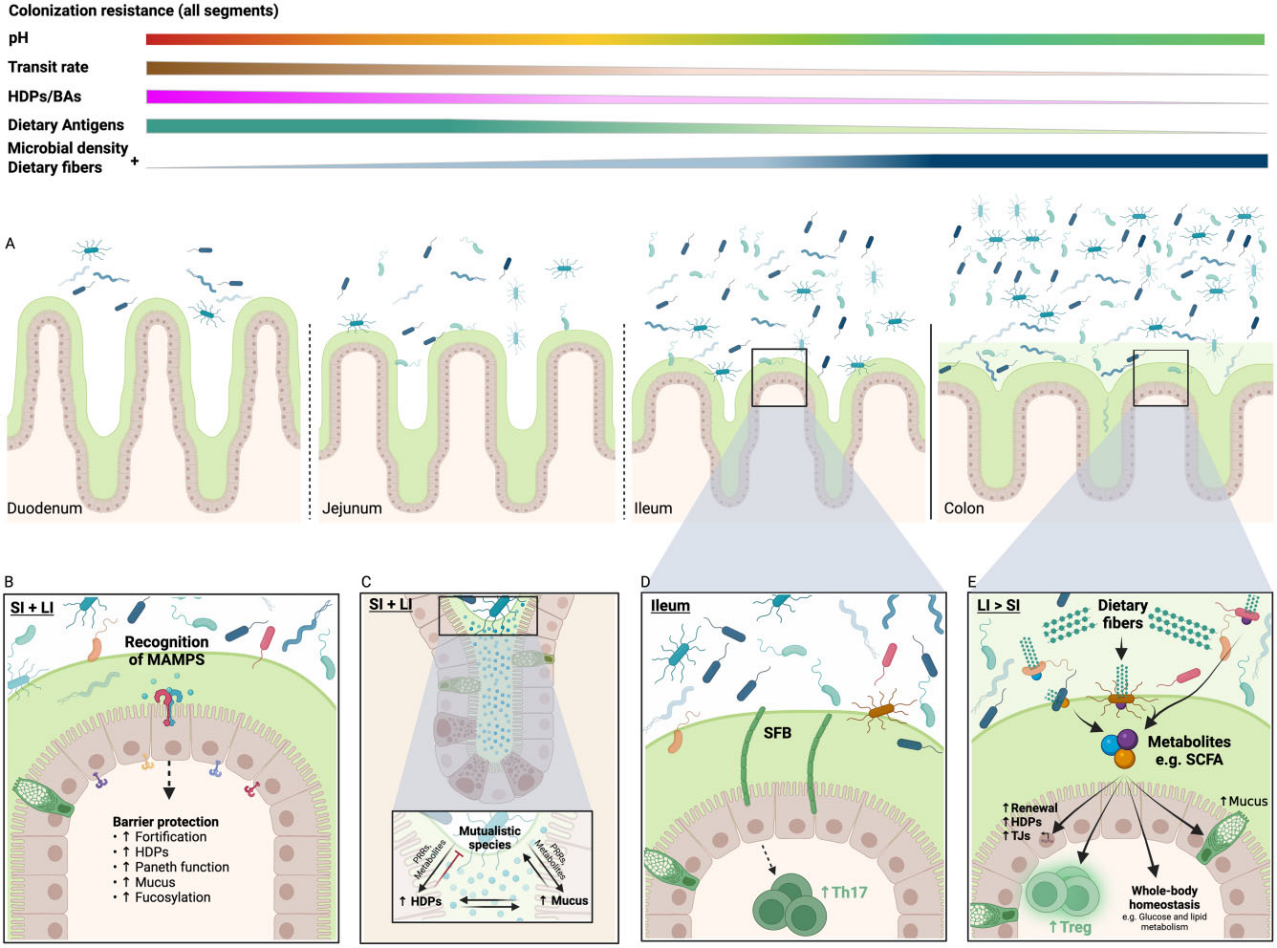
Host–microbe mutualism in the gastrointestinal tract. (A) Gastric fluid emptying into the duodenum provides an acidic environment in the proximal small intestine. pH increases along the length until it reaches neutrality in the distal ileum. High concentrations of BAs and HDPs is mirrored by low microbial load in the proximal small intestine. Dietary antigens, transit rate, BAs, and HDPs gradually decrease through the segments, thus inversely correlating with loads of indigestible fibers as well as microbial density and diversity. (B) MAMPs and metabolites are recognized by different IECs through distinct PRRs with altered expression patterns on the basolateral and the apical surface of the IEC. Broadly, MAMPs can stimulate epithelial cytokine production, promote upregulation of TJs, increase mucus production, epithelial fucosylation, HDP secretion, and maintenance of Paneth cell functions. The increased mucus production ensure that the mutualistic bacteria are confined to the mucosa, while simultaneously providing these mucolytic species with nutritional substrates for consumption. (C) HDPs protect the IECs by ensuring that the innermost layer of mucus closest to the epithelium is devoid of bacteria. (D) In mouse ileum, SFB penetrate the mucus layer and attach to the IECs. This attachment drives epithelial cytokine production and downstream increased differentiation of Th17 cells important for barrier protection. (E) Host indigestible fibers are fermented by colonic microbes and generate host-beneficial metabolites, such as SCFAs. SCFAs, along with other microbial metabolites, help strengthening the intestinal barrier through several means; increased mucus production, promotion of IEC renewal, increased production of HDPs, upregulation of TJs, and promoting regulatory T-cell differentiation. In addition, SCFAs contribute to the maintenance of whole-body homeostasis through regulation of glucose and lipid metabolism. Created with BioRender.com. SI, small intestine; LI, large intestine; HDPs, host-defense peptides; PRRs, pattern recognition receptors; MAMPs, microbe-associated molecular patterns; SFB, segmented filamentous bacteria; SCFA, short-chain fatty acids; TJs, tight junctions; BAs, bile acids; IECs, intestinal epithelial cells.

The gastrointestinal (GI) tract represents the largest surface area of the body exposed to an external environment. The intrinsic challenge on whether to respond or not respond to foreign antigens [1] is orchestrated by the mucosal immune system. Regionalized specialization is required to meet these demands in a swiftly changing landscape from the proximal duodenum to the distal colon. Change of scenery relates to different exposures to both food antigens and resident microbes. To facilitate homeostasis, the mucosal immune system should remain tolerant while preserving immunoreactivity towards invading pathogens. The colonization of amutualistic microbes is gradually increasing from the upper to the lower part of the intestine. Rapid transit time of luminal contents and low pH value as well as high concentrations of bile acids, digestive enzymes, host-defense peptides (HDPs) and immunoglobulin A (IgA) make the duodenum, jejunum, and proximal ileum unfavorable environments for bacterial growth [2]. Thus, relatively few bacteria can colonize these parts of the intestine, which are largely dominated by facultative anaerobes and acid-tolerant bacteria including *Helicobacteriaceae*, *Streptococcus*, *Enterobacteriaceae*, and *Lactobacillaceae* [3–5]. Additionally, in these regions, most simple carbohydrates, proteins, fats, and other nutrients are digested by host enzymes and absorbed. The environment of the distal ileum is characterized by a pH value close to neutral, lower concentrations of antimicrobial molecules, and increased load of dietary fibers, ultimately resulting in augmented microbial load and diversity [2, 4] (Figure 1).

The colon harbors by far the highest density of microorganisms due to a greatly reduced transit rate, low concentrations of HDPs and oxygen, as well as a scarcity of simple carbon sources, resulting in dense growth of anaerobic bacteria capable of fermenting complex dietary fibers. These microbes, primarily members of *Proteobacteria*, *Actinobacteria*, *Bacteroidaceae*, *Prevotellaceae*, *Lachnospiraceae*, and *Clostridium*, engage in prime examples of host–microbe mutualism, where the host provides a warm and nutrient-rich environment for the microbes to thrive in, while the microbiota in turn carries the enzymes needed for fiber fermentation and thereby aid in metabolizing otherwise indigestible nutrients [2, 4, 5].

Besides alterations in microbial distribution along the length of the gut, differences are also seen from the intestinal epithelial surface through the mucus layer towards the lumen [6, 7]. Colonic mucus is composed of an outer loose layer populated by bacteria and an inner more compact layer largely devoid of bacteria. The colonic outer mucus layer serves as a nutrient source for mucolytic bacteria such as *Akkermansia muciniphila* and certain members of the *Bacteroides*, *Lactobacilli*, and *Bifidobacteria* genera, while the lumen is home to members of the *Prevotellaceae*, *Bacteroidaceae*, and *Rikanellaceae* families [4, 7]. Both mucus-associated and luminal bacteria interact with the host through diffusion of microbial-produced metabolites or direct host immune interaction mediated through sampling of microbe-associated molecular patterns (MAMPs) such as lipopolysaccharide (LPS), lipid A, peptidoglycans, flagella, and microbial nucleic acids. MAMPs are recognized by pattern recognition receptors (PRRs) including Toll-like receptors (TLRs) and nuclear oligomerization domain-like receptors expressed by intestinal epithelial cells and the underlying immune cells of the lamina propria (LP). Tolerance towards luminal antigens is mounted by several means such as homeostatic cross-epithelial sampling by CX3CR1^+^ macrophages and subsequent delivery to migratory CD103^+^ dendritic cells (DCs) through gap junctions, ultimately driving differentiation of regulatory T-cells (Tregs) [8, 9]. The mucosal immune system further gauge microbial activity via direct transfer across the epithelial barrier by small intestinal Microfold-cells (M-cells) or Goblet cell-associated antigen passages (GAPs) [10]. Additionally, IgA coating of luminal bacteria facilitates transport via the polymeric Ig receptor across the epithelium. The near vicinity of the intestinal barrier to trillions of microorganisms accentuates the intimate relationship between host and microbe, and emphasizes the necessity for a well-balanced immune system to ensure tolerance against friendly residents while preserving proper immune reaction towards pathogens and opportunists [11, 12].

This review presents key aspects of host–microbe mutualism, highlights its context dependency, and describes how the mucosal immune system orchestrates appropriate immune responses towards gut microbes, be they mutualistic or pathogenic, to sustain whole-body homeostasis. Finally, we discuss promising strategies to rewire host–microbe interactions and reinstate barrier function during GI inflammation.

## Regionalized barrier defenses

The intestinal epithelium displays a high degree of heterogeneity tailored to meet the regionalized physiological requirements [13, 14]. The epithelial lining additionally functions as a protective barrier separating the internal body from foreign encounters, including innocuous antigens derived from the diet and/or the gut microbiota, as well as potentially harmful pathogens utilizing the intestines as an entry site. Accordingly, barrier defects compromise immunological tolerance facilitating excessive immune activation towards the gut microbiota, thereby fueling inflammation. To avoid this harmful trait, intestinal homeostasis is maintained by specialized barrier protective cell types. Paneth and goblet cells belong to the secretory epithelial lineage responsible for HDP and mucus production, respectively. In health, Paneth cells are restricted to the small intestine and inversely associated with microbial loads based on their HDP secretion [15], whereas the mucus-producing goblet cells are quantitatively mirrored by microbial density [16]. This trait is also reflected in regionalized mucus discrepancies between the small and large intestines (Figure 1). As such, the upper intestinal tract is covered by a single discontinuous mucus layer, allowing nutrient uptake, whereas the mucus organization in the colon is far denser. Additionally, colonic mucus consists of both an inner layer impenetrable for the gut microbiota under homeostatic conditions and an outer layer nurturing the mucus-associated bacteria, while maintaining a healthy distance between host and microbes, and likewise protecting the epithelium from mechanical damage imposed by passing stool [14, 17]. Apart from mucus secretion, small intestinal goblet cells partake in maintaining immune tolerance, in particular towards dietary antigens, through GAPs, where luminal antigens are passively sampled and delivered to the underlying migratory CD103^+^ DCs in the LP [8, 10]. Moreover, a rare sentinel goblet cell subtype residing around the colonic crypt entrance participates in barrier protection through TLR/MyD88-signaling and NACHT, leucine-rich repeat, pyrin domain-containing 6 (NLRP6) inflammasome activation towards crypt intruders by inducing compound exocytosis, thereby expelling potentially infected goblet cells and mucus, shielding the crypts from microbial colonization and invasion in mice [18]. The relevance of this mechanism is evident from NLRP6-knockout mice where generational segregation nourishes an inflammatory-prone microbiota, thus ultimately increasing the risk of intestinal inflammation and susceptibility to chemically induced colitis [19]. Subsequent studies did, however, point towards a certain degree of redundancy in this barrier protective system, as lifetime separation of littermate-controlled mice failed to develop a similar phenotype in two geographically separated cohorts [20], suggesting that multiple generations—and not maternal inheritance per se—are needed to support the gradual development of inflammation-prone microbes. As an added advantage to the NLRP6-signaling, the mentioned sentinel goblet cells further communicate through intercellular gap junctions to upper crypt goblet cells promoting enhanced mucus production, thus bolstering barrier integrity [18].

## Maintaining tolerance at the mucosal barrier

The abundant exposure to foreign antigens necessitates immune tolerance of the intestinal environment to prevent chronic inflammation [16, 21]. This is orchestrated at the inductive sites of the intestinal immune system and maintained at the effector sites [22, 23]. The inductive sites comprise the mesenteric lymph nodes (mLNs) and gut-associated lymphoid tissues (GALT), where the latter include Peyer’s patches, the cecal patch, colonic patches, isolated lymphoid follicles, and immature cryptopatches (in the mouse) [24]. The different lymphoid tissues of the gut perform specialized but overlapping functions in relation to regulating tolerance vs immune activation [25]. While numerous checkpoints are in place to ensure well-balanced immunity, a key effector arm relates to the substantial amounts of transforming growth factor β (TGFβ) produced by the epithelium. TGFβ imprints newly recruited monocytes from the bloodstream to obtain an immune-suppressive phenotype within the tissue, hallmarked by interleukin (IL)-10-responsive macrophages [22, 23]. Supportive mononuclear phagocytes include conventional DC1 (cDC1) and cDC2, phenotypically defined by expression of CD103^+^CD11b^–^ and CD103^–^CD11b^+^/CD103^+^CD11b^+^, respectively. cDC1 and cDC2 link the innate and adaptive immune systems with discrete abilities to drive appropriate effector responses [26–28]. In the steady state, migratory CD103^+^ DCs acquire antigen from the lumen and subsequently upregulate C–C chemokine receptor type 7 (CCR7) to facilitate antigen-probed DC migration from the mucosa to the mLNs following gradients of chemokine (C–C motif) ligand 19 (CCL19) and CCL21 into the afferent lymphatics [29, 30]. Both these chemokines are expressed by lymphoid-tissue-associated stromal cells [31] and secreted or transcytosed into the lumen of high endothelial venules (HEVs) [32, 33], whereas only CCL21 is expressed by lymphatic endothelial cells [33]. Thus, CCL21 primarily guides the migrating DCs to the lymph nodes, whereas both chemokines partake in guiding naive T-cells into the lymphoid tissue through HEVs, increasing the likelihood of T-cell recognition of cognate antigen [34]. CD103^+^ DCs are potent inducers of Forkhead box P3^+^ (FoxP3^+^) Tregs, as well as the expression of gut-homing molecules (CCR9 and α4β7) through their locally imprinted ability to produce retinoic acid (RA) [21, 35]. RA is derived from vitamin A metabolism, pointing towards the modulating effect of diet on intestinal immune homeostasis. This is further corroborated by a seminal report showing that depletion of vitamin A in obese mice affects T-cell trafficking, thereby aggravating obesity-induced barrier deficiencies, gut-microbiota dysbiosis, and metabolic co-morbidities [36].

The mentioned tolerogenic immune traits are compromised by barrier dysfunction, where gut-microbiota stimulation of PRRs on DCs, epithelium, mesenchymal stromal cells, and other local cell types induces pro-inflammatory cytokine expression and leukocyte recruitment, perpetuating a pro-inflammatory response and ultimately compromises the immune-suppressive state [23, 37]. Additionally, the inflammatory milieu promotes expression of co-stimulatory molecules on cDCs, enabling them to initiate an appropriately focused non-tolerogenic adaptive immune response in the draining mLN [37]. Activated antigen-specific effector T- and B-cells subsequently leave the mLN through efferent lymphatics to the thoracic duct and circulate back to the mucosa to exert their effects locally [38]. Conversely, in the absence of barrier breach and inflammation, migrating antigen-probed DCs would not express co-stimulatory molecules and thereby not orchestrate an effector response. Instead, interaction between migrating DCs and their cognate naive T-cells would, combined with RA production and the TGFβ-enriched environment, lead to the induction of *Foxp3* and gut-homing molecules, increasing the amount of intestinal Tregs [39], thus maintaining tolerance. Strategies to affect DC maturation status and/or Treg induction may therefore be actively exploited to regain tolerance in chronic inflammatory diseases.

## Host–microbe communication at the mucosal surface

Of further importance in maintaining and promoting tolerance is the presence of innocuous microbes. Here, the gut microbiota confers colonization resistance by limiting the growth of potential intruders via niche occupation, nutrient competition, and bacteriocin secretion [4, 40]. Although certain species in the interpersonal gut microbiota exhibit pathogenic potential under aberrant circumstances and are thus referred to as pathobionts, we coexist with most of our GI inhabitants in a mutually beneficial relationship. Host–microbe mutualism can, among other niches, be found at the intersection of the intestinal epithelium and mucus layer. In both humans and rodents, some bacteria such as *Bifidobacterium* and *A. muciniphila* can penetrate the mucus layer and consume its constituents [7, 41], while other bacteria, such as segmented filamentous bacteria (SFB), can directly attach to the murine epithelial cell surface of the ileum [42–44]. This interaction is essential for induction of LP CD4^+^RORγt^+^ T-helper 17 (Th17) cells in mice [43, 44] and such gut-specific Th17 cells are instrumental for metabolic homeostasis [36], supporting the mutualistic relationship between host and SFB. Both SFB and MAMPs from mucus-associated bacteria, recognized by PRRs, are part of a signaling circuit stimulating downstream production of IL-22 from group 3 innate lymphoid cells (ILC3s) [5, 45]. Rather than regulating the function of bona fide immune cells, IL-22 predominantly acts on epithelial cells located at host–environment interfaces where the target cells constitute a protective barrier as seen in the skin, liver, kidneys, and respiratory and GI tracts [46, 47]. IL-22 has been shown to alleviate high-fat diet (HFD)-induced intestinal epithelial cell stress in mice [48, 49], is important for Paneth cell function and induction of HDP expression in different tissues [47, 50–52], stimulates mucus production of murine and human goblet cells [53–55], mediates epithelial barrier fortification [45, 53], and at least in mice promotes intestinal epithelial glycan fucosylation to facilitate the growth of mutualistic mucus-associated species [56, 57]. Fucose can be used by mucolytic *Bacteroides* spp. and *A. muciniphila* as nutrient sources [58–60]. In return, these bacteria generate short-chain fatty acid (SCFA) metabolites [56, 61] exerting a wide range of beneficial effects on host physiology and homeostasis, as reviewed in detail elsewhere [62]. Interestingly, while *A. muciniphila* has received much attention as a proposed probiotic [63], emerging evidence also links this microbe to inflammatory-prone GI diseases, including graft vs host disease [64], colitis [65], and small intestinal injury [66], pointing towards context-dependent host–microbe interactions. The apparent dichotomous host responses to the same microbe also highlight why it might be more advantageous to administer either lysed bacteria or bacterial products, as effectively done with *A. muciniphila* [67, 68], *Methylococcus capsulatus* Bath (McB) [69], and *Bifidobacterium bifidum* [70], all of which curb GI inflammation by microbe-specific immune imprinting.

## Shaping the gut microbiota

### Diet

As briefly touched upon above, a prime example of host–microbe mutualism is the ability of gut microbes to metabolize indigestible dietary components such as complex fibers, for which the host lacks the necessary metabolic enzymes. The breakdown of fibers liberates carbon sources for the microorganisms to feast on and provides the host with key metabolites affecting pivotal processes of host physiology [62, 71, 72]. Diet composition is therefore one of the most prominent determinants of gut-microbiota composition and function. Disparate diets exert distinct selection pressures due to variances in substrates available for bacterial metabolism, ultimately selecting for bacteria best capable of utilizing the particular diet [73]. Human populations consuming a diet rich in fibers, such as the Hadza hunter-gatherers from Tanzania or secluded Amerindians of South America, have increased overall microbial diversity with specific enrichment of *Prevotella* compared with consumers of a Western diet (WD) that is low in fiber but high in proteins and fats [74–76]. Enrichment of *Prevotella*, particularly *Prevotella copri*, is also predictive for Western individuals responding favorably to a high-fiber, barley-kernel-based diet [77]. Paradoxically, numerous reports additionally implicate *P. copri* in inflammatory and metabolic disorders [78–80], e.g. by causally enhancing branch chain amino acid (BCAA) transport over the gut epithelium, fueling mouse and human insulin resistance [78], signifying opportunistic behaviorism of this bacterium. Although potential strain differences cannot be excluded in the above-mentioned human studies (for both *A. muciniphila* and *P. copri*), it is worth noting that the preclinical mouse studies referred to were indeed conducted with commercially available strains, matched between the diverging reports. Observed dichotomy thus corroborates microbial plasticity and presents a notable testimony of context dependency that should be carefully considered when applying live microbes, such as probiotic formulas, to human subjects [81]. Supporting this notion, a fascinating proof-of-concept study of how diet and probiotics affect the gut microbiota and clinical outcomes of melanoma checkpoint-inhibitor immunotherapy convincingly demonstrated that while high-fiber diet intake, known to diversify microbial community structures, improved treatment efficacy, patients taking over-the-counter probiotics conversely exhibited significantly poorer clinical outcomes than their none-probiotic-using counterparts [82].

The host-benefitted significance of bacterial breakdown of dietary components becomes evident considering that up to 10% of the human daily energy requirement is provided by colonic fermentation [83]. The major end-products of bacterial fermentation are SCFAs, in particular acetate, butyrate, and propionate, exhibiting beneficial effects on host health and physiology [62] including increased mucus production [84] and tight-junction assembly [85] of human colonic epithelial cells, B-cell IgA class switching through DC interactions in mice [86], and stimulating HDP expression in mice and human intestinal epithelial cells [87–89] as well as facilitating murine Treg generation through epigenetic stabilization of *Foxp3* [90–92]. When complex fibers are scarce, typical SCFA producers adapt to ferment amino acids that escaped digestion in the small intestine. This feature is particularly pronounced during WD intake, where excess proteins are metabolized by gut bacteria to BCAA and branched-chain fatty acids (BCFA), associated with insulin resistance and colonic inflammation [62, 78, 93, 94]. BCFA production is protein-specific. Accordingly, changing the protein source from casein to a protein mix resembling a typical WD for human consumption altered the murine gut microbiota to enhance the generation of BCFAs exacerbating diet-induced obesity and hepatic insulin resistance through the mTORC1/S6K1 signaling pathway [95]. Conversely, substituting the protein source in WDs from casein to whole-cell lysates of the methanotrophic soil bacterium, McB, reversed WD-induced pathogenic traits in mice [69]. The McB diet normalized the composition and functional landscape of the murine gut microbiota along with reduced fat mass, enhanced colonic mucus production, and improved glucose regulation, and promoted a consistent upregulation of FoxP3^+^RORγt^+^IL-17^+^ peripherally induced Tregs (described in further detail in the dedicated section below) [69]. Similar to changing the protein source, adding fermentable (inulin) but not insoluble (cellulose) fibers to HFDs markedly restored intestinal homeostasis in mice. In short, HFDs diminish both microbiota diversity and density, which curbed enterocyte proliferation and suppressed HDP secretion, thus enabling bacterial encroachment. Fiber enrichment ameliorated these traits in a microbiota-dependent, yet SCFA-independent, manner. Specifically, inulin-mediated microbiota-dependent restoration of IL-22 expression facilitated enhanced HDP expression, preventing microbiota encroachment and metabolic inflammation [48]. Another hallmark example of diet–microbe–host interactions relates to the intake of dietary emulsifiers—a frequently used additive smoothing visual appearances. Dietary emulsifiers have been shown to potentiate intestinal disturbances of both mice and man, including increased risk of colitis and key features of metabolic syndrome as well as intensifying the pro-inflammatory potential of gut microbes. Emulsifier-mediated alterations in the murine gut microbiota were both necessary and sufficient to drive the above-mentioned pathologies [96]. These pioneering observations have recently been confirmed in a randomized human controlled-feeding study [97], emphasizing that diet composition powerfully influences the gut-microbiota composition and function, and thus the nature of host–microbe interactions.

### HDPs

Aside from diet, the host itself has developed several mechanisms to regulate the magnitude and composition of microorganisms, populating its epithelial surfaces to maintain whole-body homeostasis. The gut microbiota and host immune system constantly crosstalk, where the host’s specific goal is to allow beneficial microorganisms to flourish yet confining them to the external environment, i.e. the intestinal lumen and outer mucus layer (Figure 1). HDPs, formerly known as antimicrobial peptides, are one of the evolutionarily oldest representations of innate immunity, found in essentially all branches of the phylogenetic tree of life [40, 98], and are central players in keeping the gut microbiota at arm’s length. They are small cationic peptides, typically <100 amino acids, and characterized by six highly conserved cysteine residues forming three disulfide bonds [99, 100]. In the gut, synthesis of HDPs is largely, but not exclusively, handled by Paneth cells at the bottom of the small intestinal crypts of Lieberkühn. In humans, Paneth cells are responsible for the production and secretion of human α-defensin 5 (HD5) and HD6, lysozyme, group 2A phospholipase A2 (PLA2G2A), and the C-type lectin Regenerating islet-derived protein 3α (REG3α), whereas other intestinal epithelial cells generally produce human β-defensin 1 (hBD1) and -2 (hBD2) [99, 101]. HDPs are largely kept within the mucus layer due to electrostatic interactions with mucin glycoconjugates, thus forming a decreasing gradient outward with higher concentrations close to the intestinal epithelium [101, 102]. This gradient protects against invading pathogens that are due to the resulting hostile environment of the inner mucus layer, while being sufficiently diluted at the outer mucus layer to maintain a symbiotic relationship with mutualistic bacteria, underscoring the important role of HDPs in controlling host–microbe interactions. While some HDPs, such as REG3α [103] and lysozyme [104, 105], confer their bactericidal properties by disrupting bacterial membrane integrity, others interfere with bacterial membrane synthesis [101] or even self-assemble into large nanonets to capture and immobilize microbes, as is the case with HD6 [106]. The host is generally protected against the deadly properties of its own HDPs, due to the cationic nature of HDPs, resulting in strong electrostatic attractions towards the negatively charged bacterial membrane and weak attractions towards the less negative eukaryotic host cell [107, 108].

The crucial role of Paneth-cell-derived HDPs in keeping mutualistic microbes in check and in regulating their composition is corroborated by studies in transgenic mouse models with aberrant Paneth cell function. Here, the gut-microbiota composition, particularly the mucus-associated microbiota, was significantly altered compared to wild-type mice [109–111]. Similarly, both genetic and diet-induced Paneth cell dysfunctions promote increased inflammatory reactions towards mutualistic bacteria, enhancing bacterial translocation and mucosal inflammation [101, 109, 112, 113]. Further supporting the relevance of HDPs in maintaining and even re-establishing homeostasis, oral administration of fungal lysozyme enhances the bacterial lysis zone near the epithelium in HFD-fed mice, thereby shielding the mucosal immune system from microbial exposure [114]. Intriguingly, microbial lysis is not exclusively beneficial and the phenotypic trait of mammalian lysozyme activity is vastly influenced by the inflammatory tone and anatomical compartment. Thus, during relapsing inflammation, as seen in inflammatory bowel disease (IBD), Paneth cells emerge in the large intestine providing increased HDP production in this compartment. Evolutionarily, such a mechanism is likely a result of colonic mucus deterioration followed by microbial invasion instigating a potent HDP response. Yet, as the microbial community structure is fundamentally different in the colon compared with the small intestine, lysozyme-mediated microbial lysis in the former might potentiate inflammatory flares. A prime example is *Ruminococcus gnavus*, a prominent member of the colonic but not the small intestinal community and therefore not affected by small intestinal Paneth-cell-secreted lysozyme in a steady state. However, during chronic inflammation, ectopic Paneth cell formation in the lower intestine exposes *R. gnavus* to endogenous lysozyme [115]. Rather than protecting the host from microbial invasion akin to the role of lysozyme in the small intestine [101], this context-dependent lysis of *R. gnavus* liberates intracellular pro-inflammatory molecules, thereby aggravating colonic disease activity [116]. This feature may be circumvented by using fungal lysozyme belonging to the glycoside hydrolase family (GH) 25 instead of the functionally distinct (GH22 family) mammalian lysozyme. Fungal lysozyme appears capable of leveraging the gut microbiota to dose-dependently alleviate experimental colitis. Diminished colitis was followed by increased colonic *R. gnavus* abundances in two genotypes, both sexes, and across three geographically and inter-continent separated facilities, pointing towards a markedly different microbiota imprinting ability of GH25 family lysozymes compared to GH22 family lysozymes [114].

One may wonder how HDPs can be one of the evolutionarily oldest and conserved systems of innate immunity yet remain highly efficient with few known mechanisms of stable antimicrobial resistance, given the highly adaptive nature of microorganisms [117, 118]. The answer may lie in the substantially complex nature of HDPs. There are currently >2,600 known varieties of HDPs [119] and this complexity can be further expanded by HDP fragmentation [120–122]. A low redox potential and naturally occurring redox enzymes create a reducing environment in the gut, known to alter the tertiary structure of HDPs by opening the disulfide bonds [123–125]. In contrast to folded oxidized peptides, such linearized forms of HDPs can be cleaved by naturally occurring proteases in the duodenal fluid [126] or by bacterial proteases [127]. Cleavage of reduced HDPs was previously believed to facilitate antimicrobial inactivation [126, 127]. A novel biological concept was therefore fostered when recent reports demonstrated how proteases present in human duodenal fluid cleaved reduced hBD1 into an active antimicrobial octapeptide [122], as well as HD5, but not HD6, into bioactive molecules with an antimicrobial repertoire exceeding that of the full-length peptide [120]. Supporting these findings, reduced human neutrophil peptide-4 (HNP-4) was similarly shown to undergo proteolytic digestion by trypsin (found in pancreatic secretions) into active antimicrobial peptide fragments capable of combating multidrug-resistant bacteria [121]. HDP fragmentation may therefore represent an evolutionary refinement meant to fine-tune the gut-microbiota composition in a changing environment.

## Rewiring gastrointestinal immunity by HDPs

Accumulating evidence further repositions HDPs as prominent immune regulators [128] both within [129] and outside [130–132] the GI tract. A recent study by Liang and colleagues [133] elegantly demonstrated that non-obese diabetic (NOD) mice exhibited diminished abundances of the HDP, cathelicidin-related antimicrobial peptide (CRAMP), associating with gut-microbiota dysbiosis. Intracolonic supplementation of CRAMP rescued colonic microbiota and immune alterations in NOD mice, thereby protecting against pancreatic autoimmunity-related diabetes [133]. While the complexity and versatile mode of action of these fascinating peptides remains under intense investigation, much of the current research points towards altered immune chemotaxis. As an example, hBD2 has repeatedly been shown to interact with chemokine receptors CCR2 [134] and CCR6 [135], thereby impacting immune cell trafficking. Direct binding to CCR2 on *in vitro* generated DCs further curbed TLR-mediated inflammation and lowered the expression of co-stimulatory molecules on responding DCs [129], suggesting that hBD2 may be used as a novel biological tool to curb DC-primed T-cell activation; it could potentially be exploited to re-establish tolerance in GI disorders. Another testimony to this potential relates to the observation that CCR2 enhances CD25 expression on FoxP3^+^ Tregs, increasing their abundance in mice independently of chemotaxis and CCR2^+^ myeloid cells (including DCs) [136]. It is therefore worth speculating that CCR2 agonists, such as hBD2, would be able to promote Treg generation or maintenance by elevated CD25 expression in FoxP3^+^ T-cells and simultaneously lower the number of effector T-cells (via diminished expression of co-stimulatory molecules on migrating DCs), collectively favoring tolerogenic immunity.

**Figure 2.**
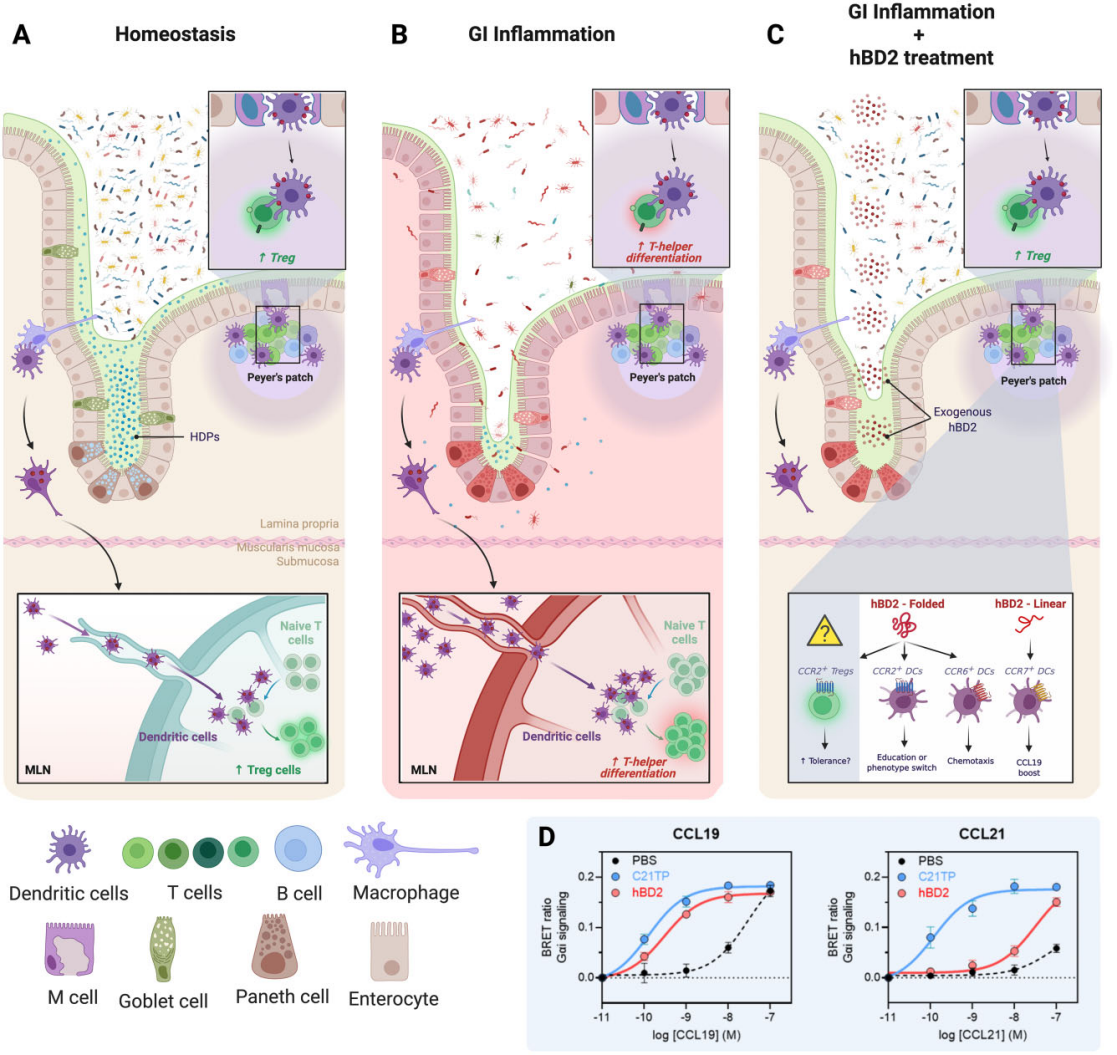
hBD2 treatment curbs gastrointestinal inflammation. (A) In steady state, the intestinal microbiota is highly diverse and largely kept in the lumen. The small intestinal crypts of Lieberkühn are kept sterile by the high concentrations of locally secreted HDPs by crypt-resident Paneth cells. Luminal antigens are sampled through several means; local CX3CR1^+^ macrophages send extensions into the mucus and lumen to sample microbes. Subsequently, sampled antigens are delivered to CD103^+^ DCs, which migrate to the mLNs to present antigen to naive T-cells. In the absence of danger signals, such interaction induces the differentiation of cognate T-cells to gut-homing Tregs. Similarly, antigens transported through M-cells in the follicle-associated epithelium covering Peyer’s patches is taken up by local underlying DCs. The DCs subsequently migrate into the T-cell zone of Peyer’s patches and induce differentiation of T-cells to Tregs in a similar manner as in mLNs. (B) During severe gastrointestinal inflammation, as seen in IBD or infectious diseases, the microbial diversity drops drastically and is largely dominated by opportunistic and pathogenic species. Paneth cell dysfunction, as can be seen in both Crohn’s disease and during HFD feeding, reduced tight-junction expression and deterioration of the mucus layer further impairs barrier integrity. Together, this allows encroachment of bacteria and increases the risk for bacterial translocation. The inflammatory environment with increased microbial-derived antigens and thus PRR stimulation induces the expression of co-stimulatory molecules and cytokine production of DCs, rendering them able to induce non-regulatory effector T-cells. (C) Administration of physiologically relevant amounts of exogenous hBD2 partially restores barrier function by promoting epithelial fortification, thereby lowering the inflammatory burden and ultimately restoring microbial diversity. Due to the low redox environment of the gut, which is exacerbated during inflammation owing to increased generation of nitrate and reactive oxygen species (ROS), HDPs are reduced and cleaved into novel antimicrobial fragments, expanding the antimicrobial repertoire. HDP—thereby also hBD2—linearization may further affect their potential to boost chemokine signaling (Panel D). Additionally, folded hBD2 have been shown to act through CCR2 and CCR6, expressed on DCs, thus facilitating chemotaxis and further partake in immune regulation. Finally, CCR2 stimulation of FoxP3^+^ Tregs boosts Treg abundances via CD25 upregulation, lending credence to the hypothesis that alternative CCR2 ligands, such as exogenously administered hBD2, can be used to as a therapeutic tool to regain tolerance. (D) Linearized hBD2 selectively enhances the activity of CCL19 over CCL21, both acting through the chemokine receptor CCR7, thereby precision editing immune chemotaxis. Experimentally, we measured CCR7 activity through Gαi as the ability to inhibit a forskolin-induced increase in intracellular cAMP. CHO cells transfected with a CCR7 plasmid and the cAMP BRET sensor CAMYEL were stimulated with various concentrations of chemokines in the presence of C21TP, hBD2 (both 10µM), or phosphate buffered saline. C21TP is a C-terminal fragment of CCL21 that boosts the activity of both CCL19 and CCL21 through CCR7 [135]. hBD2 selectively boosts CCL19, not CCL21, signaling virtually identical to C21TP. Created in BioRender.com. DC, dendritic cell; IBD, inflammatory bowel disease; HFD, high-fat diet; mLN, mesenteric lymph node; APCs, antigen presenting cells; ROS, reactive oxygen species; cAMP, cyclic adenosine monophosphate.

Instead of competing with endogenous ligands, it was recently shown that a short linear, basic peptide (proteolytically cleaved CCL21, termed C21TP) interacts directly with the N-terminus of the chemokine receptor CCR7 and substantially increases the potency of the two endogenous ligands, CCL19 and CCL21 [137]. Considering the structure of these peptides, it is reasonable to think that short alkaline peptides shield the electrostatic charges of CCR7, allowing enhanced docking of their agonists. To corroborate this hypothesis and considering the structural similarity between C21TP and hBD2, in line with the fact that most endogenous HDPs become linearized in a reduced environment as mentioned above, we assessed whether the presence or absence of linearized hBD2 would alter the chemotactic activity of CCL19 and/or CCL21 against the DC-expressed chemokine receptor, CCR7. Indeed, while C21TP uniformly boosts CCL19 and CCL21 potency, linearized hBD2 appeared to exclusively enhance CCL19-mediated chemotaxis (Figure 2). Combined, these data suggest that linearized alkaline peptides may regulate chemotaxis and migratory pace, adding yet another piece to the puzzle on how HDPs may orchestrate GI immunity.

## HDPs and barrier dysfunction in gastrointestinal inflammation

As pointed out above, the maintenance of host–microbe mutualism is essential for upholding whole-body homeostasis. An unbalanced relationship may increase contact between the host and its microbial inhabitants, enhancing the risk of bacterial translocation and susceptibility to inflammatory GI diseases. This is supported by mounting evidence linking susceptibility genes of IBD, a general umbrella term for ileal and colonic Crohn’s diseases (CD) and ulcerative colitis (UC), that encode proteins involved in maintaining a healthy epithelial barrier function and host-defense responses towards its microbiota [12, 138, 139]. The first genetic links associated with CD were those of the nucleotide-binding oligomerization domain 2 (NOD2) receptor [140, 141], an intracellular PRR recognizing bacterial peptidoglycans [141]. NOD2 is expressed by both human and murine Paneth cells and plays a crucial role in regulating gut-microbiota composition by controlling the expression and secretion of HDPs [111, 142, 143]. NOD2 gene variants remain the strongest known risk factors for development of ileal CD [140, 141], supporting that impaired Paneth cell function and HDP production are highly implicated in at least small intestinal CD pathology [144]. These observations indicate a pivotal role for Paneth cells and their HDPs in intestinal health and that the collapse of host defenses, and thus host–microbe mutualism, plays an important role in GI inflammatory disorders. This is additionally supported by observations that loss-of-function mutations in the autophagy-related 16-like 1 (ATG16L1) protein, another CD-associated risk gene, result in impaired exocytosis of secretory granules in Paneth cells [145] and dysregulate IL-22 signaling, resulting in aggravated inflammatory responses and necrosis of intestinal epithelial cells in mice [146].

Although dysbiosis appears to be an integrated, and often necessary, part of GI inflammation, it remains debated whether gut dysbiosis is causally implicated in these multifactorial pathologies. Transfer of microbiota from human CD and UC patients to germ-free mice reduced abundances of RORγt^+^ Tregs compared with transfer from healthy controls [147], demonstrating that inflammatory proficient microbes affect host immunity. Still, immunological rewiring depended on the genetic susceptibility of the epithelial barrier dysfunction to precipitate spontaneous inflammation [147], pointing towards barrier defects as a primary cause of IBD. Along these lines, gut bacteria have been shown to translocate from the intestinal barrier into mLNs, blood, and adipose tissues following induced colitis and ileitis [148, 149]. Creeping fat is characterized by the migration and subsequent accumulation of mesenteric adipocytes clustering around inflamed and fibrotic intestinal tissues observed in patients with ileal CD [150] and is suggested to occur as a response to gut-barrier dysfunction and bacterial translocation [151], possibly serving as a protective mechanism deployed to limit the systemic distribution of potentially harmful microbes and associated molecules. This is further supported by a recent study demonstrating that bacteria isolated from creeping fat in patients with CD were able to translocate to mesenteric adipose tissue. Additionally, these bacteria were able to exacerbate colitis upon transfer to antibiotic-treated mice [152], thus lending credence to the hypothesis of creeping fat being a defense mechanism confining invading bacteria, thereby limiting systemic dissemination.

## Bacterial translocation as a function of diet-induced barrier defects

Bacterial translocation not only occurs in classical IBDs, but also in obesity [153, 154], fatty liver disease [155, 156], and other metabolic conditions characterized by a leaky gut [11, 157, 158]. It remains debated whether gut leakiness is primarily microbiota-, diet-, or disease-mediated. While gut microbes can both translocate and promote barrier breach, these are—to the best of our knowledge—often secondary hits caused by physiological disturbances, as exemplified above with dietary emulsifiers facilitating microbiota invasion [159]. Diet composition may also alter the intestinal landscape compromising host defenses. Murine WD feeding induces Paneth cell defects [112] associated with lower HDP secretion. Conversely, therapeutic treatment with physiologically relevant levels of the hallmark human Paneth cell HDPs, particularly HD5, mitigated insulin resistance and dyslipidemia in diet-induced obese mice [160], highlighting the importance of intact host-defense mechanisms in maintaining organismal homeostasis. Obesity and WD consumption predispose to hyperglycemia—a condition that independently drives barrier dysfunction, thereby enhancing colitis susceptibility and risk of enteric infections through transcriptional reprogramming of intestinal epithelial cells and tight-junction integrity [161]. Although leaky gut is associated with chronic low-grade inflammation often seen in individuals with metabolic diseases [162], reports of microbial signatures in extra-intestinal tissues have often been discredited as a result of environmental contamination. Challenging the previous dogma of sterile internal organs, multiple recent studies have used strict contamination-aware approaches to substantiate that bacteria and/or bacterial products can indeed be found in our internal organs during disease [153, 154] and even postprandial in healthy young men [155]. Corroborating biological relevance and not merely a tale of wishful thinking, bacterial abundance followed the expected anatomical route and the composition discriminated between disease states [154]. Predominantly members of the opportunistic phylum *Proteobacteria* can be found in adipose tissues [153, 154] and livers [154] of individuals with morbid obesity and type 2 diabetes. Mechanistically, microbiota-derived products from an impaired gut barrier can be transported to distal tissues by gut microbial DNA-containing extracellular vesicles (mEVs) [163]. Extracellular vesicles are normally filtered by liver CRIg^+^ macrophages and the adoptive transfer of mEVs to CRIg^–/–^ mice potentiated microbial-derived product transport to distal tissues contributing to obesity-associated tissue inflammation and insulin resistance [163]. As macrophage function is generally hampered by obesity [164] and because insulin resistance promotes hyperglycemia, it is foreseeable that this condition provides the perfect storm for initial barrier breach followed by bacterial and/or bacterial product translocation. In support, Cani and colleagues [165] originally defined metabolic endotoxemia as unusual levels of circulating bacterial endotoxins, in particular LPS, fueling obesity and insulin resistance, thus initiating a vicious immunometabolic cycle from the gut to extra-intestinal tissues. Elevated levels of LPS can be detected in the plasma of humans showing features of metabolic syndrome, including obesity, insulin resistance, dyslipidemia, and chronic low-grade inflammation [166], which is exacerbated upon high fat intake [167]. Still, as LPS is a complex molecule found in different acetylation forms affecting molecular structure [168] and thereby binding capacity, not all types of LPS are metabolically detrimental to the host. Comparing the metabolic effects of *Escherichiacoli*-derived and *Rhodobacter sphaeroides*-derived LPS, it was recently demonstrated that only *E. coli* LPS mediated intestinal barrier disruption, dysglycemia, and low-grade inflammation of adipose tissue [169]. Interestingly, rather than being a biologically neutral LPS form, *R. sphaeroides* LPS counteracted *E. coli* LPS-induced metabolic dysregulations and improved glucose metabolism in obese mice [169]. This study is therefore an elegant demonstration of the incredible context-dependent nature of host–microbe interactions and a testimony to the importance of distinguishing between LPS composition and characteristics when discussing metabolic endotoxemia.

## Rewiring gastrointestinal immunity by nutritional imprinting

As barrier defects promote unwarranted immunity towards otherwise mutualistic and thus harmless microbes, it is key to understand how we can regain immunological tolerance, towards the gut microbiota, but equally importantly toward dietary antigens. Here, acquired immunity, and most notably Tregs, is of immense importance. Two major types of FoxP3^+^ Tregs exist: natural thymic-derived FoxP3^+^RORγt^–^Helios^+^ (nTregs) and peripherally induced FoxP3^+^RORγt^–^Helios^–^ and FoxP3^+^RORγt^+^Helios^–^ (iTregs) [170]. FoxP3^+^RORγt^+^ iTregs are microbially induced, primarily in the large intestine, and have been shown to exhibit superior immune-suppressive capacity compared with anTregs, and are therefore essential for peripheral immune homeostasis [171, 172]. A key factor in peripheral induction of iTregs is RA stimulation upon antigen recognition by migratory CD103^+^ DCs in GALT and mLN [39]. During this process, other environmental factors might additionally enhance the induced FoxP3 phenotype, such as microbial bile acid metabolites [173], SCFAs [92], MyD88-signaling [174], TGFβ [39], and supportive cytokines [175–177]. While nTregs dominate the small intestinal LP, a compartment characterized by low microbial loads, FoxP3^+^RORγt^+^ iTregs are more abundant in LP of the more densely microbially populated large intestine [171]. Interestingly, tolerance was recently shown to anatomically compartmentalize, as proximal murine mLNs provided a superior tolerogenic environment compared with more distal mLNs (ileal and colonic), potentially as a consequence of the larger RA production in the upper small intestine due to higher exposure to diet-derived vitamin A [178]. This elegantly demonstrates alternative ways of compartmentalizing and maintaining intestinal tolerance in diverse intestinal environments, where proximal draining mLNs are generally associated with tolerance induction towards dietary antigens and distally draining mLNs towards the microbiota, albeit with a skewing towards easier induction of inflammation in the latter [176, 178]. Instant transition between tolerogenic immunity and inflammation may represent an evolutionary conserved trait installed to empower the host to swiftly mount a protective immune response upon barrier disruption in the more densely populated lower intestine.

**Figure 3.**
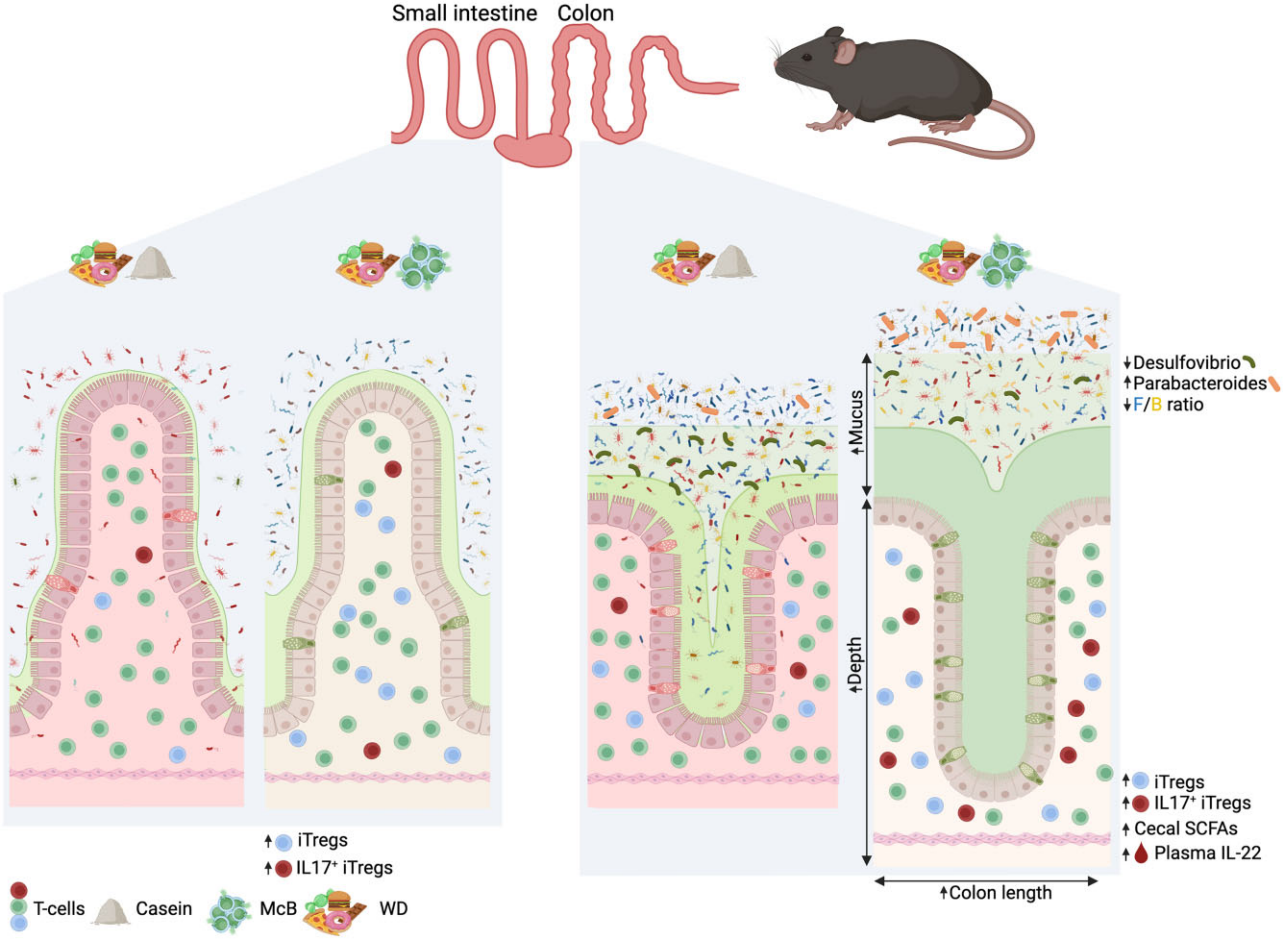
McB feeding rescues WD-induced gastrointestinal malfunctions. Small intestines (left panels) and colons (right panels) from casein- or McB-supplemented WD-fed mice. Small intestines from WD-McB-fed mice exhibited increased amounts of FoxP3^+^RORγt^+^ iTregs and augmented total amounts of IL-17-expressing iTregs compared to WD-casein-fed mice. Colons from WD-casein-fed mice (right panels) harbored considerably more *Desulfovibrio* compared to WD-McB-fed mice, which instead boosted the abundance of the health-associated *Parabacteroides*, as well as an overall decrease in the *Firmicutes*/*Bacteroides* (F/B) ratio, likewise associated with a healthy phenotype. Additionally, the middle segment of the colons from WD-McB-fed mice showed goblet cell hyperplasia and increased mucus production, specifically of neutral mucins and sulfomucins, compared to WD-casein-fed mice, suggesting improved barrier function in WD-McB-fed mice. Furthermore, the middle segment of colons from McB-fed mice had increased crypt depth and these colons also had an overall increased length, which is reciprocally associated with inflammation. Created in BioRender.com. McB, *Methylococcus capsulatus* Bath; WD, Western diet.

Specific bacterial components can induce tolerogenic responses and mitigate metabolic and GI diseases. This notion is supported by findings showing that colonic, but not small intestinal, FoxP3^+^RORγt^+^ iTregs can be effectively induced by cell surface polysaccharides of *B. bifidum* via TLR2-expressing DCs [70]. Likewise, both lysed *A. muciniphila* and a purified cell-wall component of the bacterium can mitigate metabolic dysregulation, reduce fat mass, and improve dyslipidemia in mice more potently than the live bacterium [67]. Additionally, supplementation of lysed *A. muciniphila* in human subjects improved insulin sensitivity and reduced total plasma cholesterol and fat mass compared with placebo controls in a randomized, double-blinded human study [68], thus suggesting that microbial components rather than live microbes can be exploited as novel tools to curb microbial dysbiosis associated with metabolic and inflammatory GI disorders. In agreement with both this notion and the previous studies mentioned above, bacterial lysates of McB used as a protein source in a high-fat/high-sugar WD increased the FoxP3^+^RORγt^+^ iTreg pool in both murine small and large intestines [69]. To the best of our knowledge, McB feeding represents a unique dietary intervention to promote small intestinal FoxP3^+^RORγt^+^ iTreg induction and thus holds great potential as a therapeutic strategy targeting small intestinal diseases, including ileal CD and food allergies. A large fraction of these FoxP3^+^RORγt^+^ iTregs additionally exhibited increased IL-17 production in the large intestine compared with non-McB-supplemented WD and low-fat diet (LFD)-fed mice; it is a trait that tended to persist in the small intestine [69]. The involvement and importance of Tregs to maintain intestinal immune homeostasis and the apparent importance of IL-17 in maintaining a healthy microbiota and proper barrier defense could spur the idea that augmented IL-17-producing FoxP3^+^RORγt^+^ iTregs represent a safe path to enhanced IL-17 production without additionally increasing the risk of Th17-mediated inflammation-associated pathology. Induction of FoxP3^+^RORyt^+^ Tregs in the setting of McB feeding was further associated with a remarkable change in the gut microbiota towards that of LFD-fed reference mice, despite the McB-fed mice being maintained on a high-fat/high-sugar WD [69]. Although most changes reflected those observed in LFD-fed mice, two genera appeared to be unequally affected, namely a notable increase in *Parabacteroides* mirrored by an equally dramatic decrease in *Desulfovibrio*. A reciprocal relationship was seen in WD-fed mice (standard protein source). WD-McB-induced *Parabacteroides* bloom depended on adaptive immunity. Accordingly, *Rag*^–/–^ mice exhibited negligible and uniform loads of this genus between diets. Interestingly, *Parabacteroides distasonis* both alleviates obesity and metabolic dysfunction [179] as well as chemically induced inflammation [180], akin to the effects of McB feeding [69, 181]. Conversely, *Desulfovibrio* is often increased in human colitis [182]. It promotes obesity in mice following T-cell impairments [183] and disrupts barrier function via sulfate-reducing activities [184], thereby degrading health promoting sulfomucins [185]. The sulfate-reducing activities are further proposed to be directly involved in the pathogenesis of UC by hydrogen sulfide formation [186]. WD-McB feeding significantly abrogated these traits (Figure 3).

## Concluding remarks

Dietary components and opportunistic members of the gut microbiota can both individually and synergistically exploit the intestinal immune system to overcome GI host defenses compromising barrier function. The global prevalence of GI disorders is steadily increasing, hence representing a significant—and escalating—challenge to healthcare systems, and negatively impacting life quality across socioeconomic statuses. Novel therapies targeting barrier defects are thus urgently needed. While oral administration of live microorganisms holds the potential for re-establishing GI homeostasis when being tailored to specific dietary regimens, they also come with a risk of potential side effects in subjects either not adhering to provided dietary guidelines or with increased susceptibility to specific diseases and/or microbiota-dependent treatment modalities. An alternative and more controllable path forward may therefore lay in the use of microbially derived products and/or naturally occurring HDPs being tailored to regain tolerance. However, as the barrier defect must be repaired to maintain tolerogenic immunity, we still need innovative strategies to identify intestinotrophic drug candidates. Lastly, therapies aiming at unarming opportunistic bacteria via precision editing of the gut microbiota may be of particular interest enabling the maintenance of mutualistic microbes while exclusively targeting potential invaders, thereby mitigating bacterial translocation.

## Authors’ Contributions

B.A.H.J. conceived of the idea for the manuscript. S.K.J. and S.I.P. drafted the first version with input from E.F.B., A.S.J. and G.M.H. E.P.B. and A.S.J. performed the experiments for Figure 2D under the supervision of G.M.H. S.K.J. and S.I.P. designed the figures. B.A.H.J. supervised all parts of the process. All authors read, edited, and approved the final manuscript.

## Funding

This study was supported by the Novo Nordisk Foundation [grant number: NNF17OC0026698].

## Conflict of interest

B.A.H.J. is co-inventor of International (PCT) Patent Application (No. PCT/EP2018/071076) jointly owned by University of Copenhagen, Denmark, and Norwegian University of Life Sciences, Norway, based on data related to *Methylococcus capsulatus* Bath. The remaining authors declare no competing interests.

## References

[1] Mowat AM. To respond or not to respond—a personal perspective of intestinal tolerance. Nat Rev Immunol 2018;18:405–15. 29491358 10.1038/s41577-018-0002-x

[2] Donaldson GP , Lee SM, Mazmanian SK. Gut biogeography of the bacterial microbiota. Nat Rev Microbiol 2016;14:20–32. 26499895 10.1038/nrmicro3552 PMC4837114

[3] Seedorf H , Griffin NW, Ridaura VK et al Bacteria from diverse habitats colonize and compete in the mouse gut. Cell 2014;159:253–66. 25284151 10.1016/j.cell.2014.09.008 PMC4194163

[4] Parker A , Lawson MAEE, Vaux L et al Host-microbe interaction in the gastrointestinal tract. Environ Microbiol. 2018;20:2337–53. 28892253 10.1111/1462-2920.13926 PMC6175405

[5] Brown EM , Sadarangani M, Finlay BB. The role of the immune system in governing host-microbe interactions in the intestine. Nat Immunol. 14: 2013; 660–7. 23778793 10.1038/ni.2611

[6] Daniel N , Lecuyer E, Chassaing B. Host/microbiota interactions in health and diseases—time for mucosal microbiology! Mucosal Immunol. 2021;14:1006–16. 33772148 10.1038/s41385-021-00383-w PMC8379076

[7] Li H , Limenitakis JP, Fuhrer T et al The outer mucus layer hosts a distinct intestinal microbial niche. Nat Commun. 2015;6:8292. 26392213 10.1038/ncomms9292 PMC4595636

[8] Mazzini E , Massimiliano L, Penna G et al Oral tolerance can be established via gap junction transfer of fed antigens from CX3CR1+ macrophages to CD103+ dendritic cells. Immunity 2014;40:248–61. 24462723 10.1016/j.immuni.2013.12.012

[9] Schulz O , Jaensson E, Persson EK et al Intestinal CD103+, but not CX3CR1+, antigen sampling cells migrate in lymph and serve classical dendritic cell functions. J Exp Med 2009;206:3101–14. 20008524 10.1084/jem.20091925 PMC2806467

[10] McDole JR , Wheeler LW, McDonald KG et al Goblet cells deliver luminal antigen to CD103+ dendritic cells in the small intestine. Nature 2012;483:345–9. 22422267 10.1038/nature10863 PMC3313460

[11] Zheng D , Liwinski T, Elinav E. Interaction between microbiota and immunity in health and disease. Cell Res 2020;30:492–506. 32433595 10.1038/s41422-020-0332-7 PMC7264227

[12] Caruso R , Lo BC, Nunez G. Host-microbiota interactions in inflammatory bowel disease. Nat Rev Immunol 2020;20:411–26. 32005980 10.1038/s41577-019-0268-7

[13] Parikh K , Antanaviciute A, Fawkner-Corbett D et al Colonic epithelial cell diversity in health and inflammatory bowel disease. Nature 2019;567:49–55. 30814735 10.1038/s41586-019-0992-y

[14] Barker N. Adult intestinal stem cells: critical drivers of epithelial homeostasis and regeneration. *Nat Rev Mol Cell Biol* 2013;15:19–33. 10.1038/nrm3721 24326621

[15] Ostaff MJ , Stange EF, Wehkamp J. Antimicrobial peptides and gut microbiota in homeostasis and pathology. EMBO Mol Med 2013;5:1465–83. 24039130 10.1002/emmm.201201773 PMC3799574

[16] Mowat AM , Agace WW. Regional specialization within the intestinal immune system. Nat Rev Immunol 2014;14:667–85. 25234148 10.1038/nri3738

[17] Johansson M. E V , Hansson GC. Immunological aspects of intestinal mucus and mucins. Nat Rev Immunol 2016;16:639–49. 27498766 10.1038/nri.2016.88 PMC6435297

[18] Birchenough GMH , Nyström EEL, Johansson M. E V, Hansson GC. A sentinel goblet cell guards the colonic crypt by triggering Nlrp6-dependent Muc2 secretion. https://www.science.org (2 March 2022, date last accessed). 10.1126/science.aaf7419 PMC5148821 27339979

[19] Elinav E , Strowig T, Kau AL et al NLRP6 inflammasome regulates colonic microbial ecology and risk for colitis. Cell 2011;145:745–57. 21565393 10.1016/j.cell.2011.04.022 PMC3140910

[20] Mamantopoulos M , Ronchi F, Van Hauwermeiren F et al Nlrp6- and ASC-dependent inflammasomes do not shape the commensal gut microbiota composition. Immunity 2017;47:339–48.e4. 28801232 10.1016/j.immuni.2017.07.011

[21] Pabst O , Mowat AM. Oral tolerance to food protein. Mucosal Immunol 2012;5:232–9. 22318493 10.1038/mi.2012.4 PMC3328017

[22] Zigmond E , Bernshtein B, Friedlander G et al Macrophage-restricted interleukin-10 receptor deficiency, but not IL-10 deficiency, causes severe spontaneous colitis. Immunity 2014;40:720–33. 24792913 10.1016/j.immuni.2014.03.012

[23] Mowat AM , Scott CL, Bain CC. Barrier-tissue macrophages: functional adaptation to environmental challenges. Nat Med 2017;23:1258–70. 29117177 10.1038/nm.4430

[24] Brandtzaeg P , Kiyono H, Pabst R et al Terminology: nomenclature of mucosa-associated lymphoid tissue. Mucosal Immunol 2008;1:31–7. 19079158 10.1038/mi.2007.9

[25] Luciani C , Hager FT, Cerovic V et al Dendritic cell functions in the inductive and effector sites of intestinal immunity. Mucosal Immunol 2022;15:40–50. 34465895 10.1038/s41385-021-00448-w

[26] Luda KM , Joeris T, Persson EK et al IRF8 transcription-factor-dependent classical dendritic cells are essential for intestinal T cell homeostasis. Immunity 2016;44:860–74. 27067057 10.1016/j.immuni.2016.02.008

[27] Mayer JU , Demiri M, Agace WW et al Different populations of CD11b+ dendritic cells drive Th2 responses in the small intestine and colon. Nat Commun 2017;8:15820. 28598427 10.1038/ncomms15820 PMC5472728

[28] Persson EK , Uronen-Hansson H, Semmrich M et al IRF4 transcription-factor-dependent CD103+CD11b+ dendritic cells drive mucosal T helper 17 cell differentiation. Immunity 2013;38:958–69. 23664832 10.1016/j.immuni.2013.03.009

[29] Bekiaris V , Persson EK, Agace WW. Intestinal dendritic cells in the regulation of mucosal immunity. Immunol Rev 2014;260:86–101. 24942684 10.1111/imr.12194

[30] Girard J-P , Moussion C, Förster R. HEVs, lymphatics and homeostatic immune cell trafficking in lymph nodes. Nat Rev Immunol 2012;12:762–73. 23018291 10.1038/nri3298

[31] Luther SA , Tang HL, Hyman PL et al Coexpression of the chemokines ELC and SLC by T zone stromal cells and deletion of the ELC gene in the plt/plt mouse. Proc Natl Acad Sci USA 2000;97:12694–9. 11070085 10.1073/pnas.97.23.12694 PMC18826

[32] Carlsen HS , Haraldsen G, Brandtzaeg P et al Disparate lymphoid chemokine expression in mice and men: no evidence of CCL21 synthesis by human high endothelial venules. Blood 2005;106:444–6. 15863780 10.1182/blood-2004-11-4353

[33] Gunn MD , Tangemann K, Tam C et al A chemokine expressed in lymphoid high endothelial venules promotes the adhesion and chemotaxis of naive T lymphocytes. Proc Natl Acad Sci USA 1998;95:258–63. 9419363 10.1073/pnas.95.1.258 PMC18193

[34] Förster R , Davalos-Misslitz AC, Rot A. CCR7 and its ligands: balancing immunity and tolerance. Nat Rev Immunol 2008;8:362–71. 18379575 10.1038/nri2297

[35] Iwata M , Hirakiyama A, Eshima Y et al Retinoic acid imprints gut-homing specificity on T cells. Immunity 2004;21:527–38. 15485630 10.1016/j.immuni.2004.08.011

[36] Hong C-P , Park A, Yang B-G et al Gut-specific delivery of T-helper 17 cells reduces obesity and insulin resistance in mice. Gastroenterology 2017;152:1998–2010. 28246016 10.1053/j.gastro.2017.02.016

[37] Joeris T , Müller-Luda K, Agace WW et al Diversity and functions of intestinal mononuclear phagocytes. Mucosal Immunol 2017;10:845–64. 28378807 10.1038/mi.2017.22

[38] Mowat AM. Anatomical basis of tolerance and immunity to intestinal antigens. Nat Rev Immunol 2003;3:331–41. 12669023 10.1038/nri1057

[39] Mucida D , Park Y, Kim G et al Reciprocal T H 17 and regulatory T cell differentiation mediated by retinoic acid. Science 2007;317:256–60. 17569825 10.1126/science.1145697

[40] Hassan M , Kjos M, Nes IF et al Natural antimicrobial peptides from bacteria: characteristics and potential applications to fight against antibiotic resistance. J Appl Microbiol 2012;113:723–36. 22583565 10.1111/j.1365-2672.2012.05338.x

[41] Fang J , Wang H, Zhou Y et al Slimy partners: the mucus barrier and gut microbiome in ulcerative colitis. Exp Mol Med 2021;53:772–87. 34002011 10.1038/s12276-021-00617-8 PMC8178360

[42] Jonsson H , Hugerth LW, Sundh J et al Genome sequence of segmented filamentous bacteria present in the human intestine. Commun Biol 2020;3:485. 32887924 10.1038/s42003-020-01214-7 PMC7474095

[43] Ivanov II , Atarashi K, Manel N et al Induction of intestinal Th17 cells by segmented filamentous bacteria. Cell 2009;139:485–98. 19836068 10.1016/j.cell.2009.09.033 PMC2796826

[44] Wang Y , Yin Y, Chen X et al Induction of intestinal Th17 cells by Flagellins from segmented filamentous bacteria. Front Immunol 2019;10:2750. 31824516 10.3389/fimmu.2019.02750 PMC6883716

[45] Keir M , Yi Y, Lu T et al The role of IL-22 in intestinal health and disease. J Exp Med 2020;217:e20192195. 32997932 10.1084/jem.20192195 PMC7062536

[46] Sabat R , Ouyang W, Wolk K. Therapeutic opportunities of the IL-22-IL-22R1 system. Nat Rev Drug Discov 2014;13:21–38. 24378801 10.1038/nrd4176

[47] Wolk K , Kunz S, Witte E et al IL-22 increases the innate immunity of tissues. Immunity 2004;21:241–54. 15308104 10.1016/j.immuni.2004.07.007

[48] Zou J , Chassaing B, Singh V et al Fiber-mediated nourishment of gut microbiota protects against diet-induced obesity by restoring IL-22-mediated colonic health. Cell Host Microbe 2018;23:41–53.e4. 29276170 10.1016/j.chom.2017.11.003 PMC6005180

[49] Gulhane M , Murray L, Lourie R et al High fat diets induce colonic epithelial cell stress and inflammation that is reversed by IL-22. Sci Rep 2016;6:28990. 27350069 10.1038/srep28990 PMC4924095

[50] Brand S , Beigel F, Olszak T et al IL-22 is increased in active Crohn’s disease and promotes proinflammatory gene expression and intestinal epithelial cell migration. Am J Physiol Gastrointest Liver Physiol 2006;290:G827–38. 16537974 10.1152/ajpgi.00513.2005

[51] Zheng Y , Valdez PA, Danilenko DM et al Interleukin-22 mediates early host defense against attaching and effacing bacterial pathogens. Nat Med 2008;14:282–9. 18264109 10.1038/nm1720

[52] Gaudino SJ , Beaupre M, Lin X et al IL-22 receptor signaling in Paneth cells is critical for their maturation, microbiota colonization, Th17-related immune responses, and anti-Salmonella immunity. Mucosal Immunol 2021;14:389–401. 33060802 10.1038/s41385-020-00348-5 PMC7946635

[53] Laursen MF , Sakanaka M, von Burg N et al Bifidobacterium species associated with breastfeeding produce aromatic lactic acids in the infant gut. Nat Microbiol 2021;6:1367–82. 34675385 10.1038/s41564-021-00970-4 PMC8556157

[54] Sugimoto K , Ogawa A, Mizoguchi et al IL-22 ameliorates intestinal inflammation in a mouse model of ulcerative colitis. J Clin Invest 2008;118:534–44. 18172556 10.1172/JCI33194 PMC2157567

[55] Turner JE , Stockinger B, Helmby H. IL-22 mediates goblet cell hyperplasia and worm expulsion in intestinal helminth infection. PLoS Pathog 2013;9:e1003698. 24130494 10.1371/journal.ppat.1003698 PMC3795034

[56] Pickard JM , Maurice CF, Kinnebrew MA et al Rapid fucosylation of intestinal epithelium sustains host-commensal symbiosis in sickness. Nature 2014;514:638–41. 25274297 10.1038/nature13823 PMC4214913

[57] Sonnenberg GF , Monticelli LA, Alenghat T et al Innate lymphoid cells promote anatomical containment of lymphoid-resident commensal bacteria. Science 2012;336:1321–5. 22674331 10.1126/science.1222551 PMC3659421

[58] Ottman N , Davids M, Suarez-Diez M et al Genome-scale model and omics analysis of metabolic capacities of Akkermansia muciniphila reveal a preferential mucin-degrading lifestyle. Appl Environ Microbiol 2017;83:e01014–17. 28687644 10.1128/AEM.01014-17 PMC5583483

[59] Reichardt N , Duncan SH, Young P et al Phylogenetic distribution of three pathways for propionate production within the human gut microbiota. ISME J 2014;8:1323–35. 24553467 10.1038/ismej.2014.14 PMC4030238

[60] Hooper L. V , Xu J, Falk PG et al A molecular sensor that allows a gut commensal to control its nutrient foundation in a competitive ecosystem. Proc Natl Acad Sci USA 1999;96:9833–8. 10449780 10.1073/pnas.96.17.9833 PMC22296

[61] Venegas DP , De la Fuente MK, Landskron G et al Short chain fatty acids (SCFAs)-mediated gut epithelial and immune regulation and its relevance for inflammatory bowel diseases. Front Immunol 2019;10:277. 30915065 10.3389/fimmu.2019.00277 PMC6421268

[62] Koh A , de Vadder F, Kovatcheva-Datchary P et al From dietary fiber to host physiology: short-chain fatty acids as key bacterial metabolites. Cell 2016;165:1332–45. 27259147 10.1016/j.cell.2016.05.041

[63] Cani PD. Human gut microbiome: hopes, threats and promises. Gut 2018;67:1716–25. 29934437 10.1136/gutjnl-2018-316723 PMC6109275

[64] Shono Y , Docampo MD, Peled JU et al Increased GVHD-related mortality with broad-spectrum antibiotic use after allogeneic hematopoietic stem cell transplantation in human patients and mice. Sci Transl Med 2016;8:339ra71. 10.1126/scitranslmed.aaf2311 PMC4991773 27194729

[65] Seregin SS , Golovchenko N, Schaf B et al NLRP6 protects Il10(–/–) mice from colitis by limiting colonization of Akkermansia muciniphila. Cell Rep 2017;19:733–45. 28445725 10.1016/j.celrep.2017.03.080 PMC5528001

[66] Yoshihara T , Oikawa Y, Kato T et al The protective effect of Bifidobacterium bifidum G9-1 against mucus degradation by Akkermansia muciniphila following small intestine injury caused by a proton pump inhibitor and aspirin. Gut Microbes 2020;11:1385–404. 32515658 10.1080/19490976.2020.1758290 PMC7527075

[67] Plovier H , Everard A, Druart C et al A purified membrane protein from Akkermansia muciniphila or the pasteurized bacterium improves metabolism in obese and diabetic mice. Nat Med 2017;23:107–13. 27892954 10.1038/nm.4236

[68] Depommier C , Everard A, Druart C et al Supplementation with Akkermansia muciniphila in overweight and obese human volunteers: a proof-of-concept exploratory study. Nat Med 2019;25:1096–103. 31263284 10.1038/s41591-019-0495-2 PMC6699990

[69] Jensen BAH , Holm JB, Larsen IS et al Lysates of Methylococcus capsulatus Bath induce a lean-like microbiota, intestinal FoxP3(+)RORgammat(+)IL-17(+) Tregs and improve metabolism. Nat Commun 2021;12:1093. 33597537 10.1038/s41467-021-21408-9 PMC7889900

[70] Verma R , Lee C, Jeun E-J et al Cell surface polysaccharides of Bifidobacterium bifidum induce the generation of Foxp3 + regulatory T cells. Sci Immunol 2018;3:eaat6975. 10.1126/sciimmunol.aat6975 30341145

[71] Wu Y , Wan J, Choe U et al Interactions between food and gut microbiota: impact on human health. Annu Rev Food Sci Technol 2019;10:389–408. 30908952 10.1146/annurev-food-032818-121303

[72] Gentile CL , Weir TL. The gut microbiota at the intersection of diet and human health. Science 2018;362:776–80. 30442802 10.1126/science.aau5812 PMC13264711

[73] Ecklu-Mensah G , Gilbert J, Devkota S. Dietary selection pressures and their impact on the gut microbiome. Cell Mol Gastroenterol Hepatol 13: 2022;7–18. 34329765 10.1016/j.jcmgh.2021.07.009 PMC8600059

[74] Clemente JC , Pehrsson EC, Blaser MJ et al The microbiome of uncontacted Amerindians. Sci Adv 2015;1:e1500183. 26229982 10.1126/sciadv.1500183 PMC4517851

[75] Yatsunenko T , Rey FE, Manary MJ et al Human gut microbiome viewed across age and geography. Nature 2012;486:222–7. 22699611 10.1038/nature11053 PMC3376388

[76] Schnorr SL , Candela M, Rampelli S et al Gut microbiome of the Hadza hunter-gatherers. Nat Commun 2014;5:3654. 24736369 10.1038/ncomms4654 PMC3996546

[77] Kovatcheva-Datchary P , Nilsson A, Akrami R et al Dietary fiber-induced improvement in glucose metabolism is associated with increased abundance of prevotella. Cell Metab 2015;22:971–82. 26552345 10.1016/j.cmet.2015.10.001

[78] Pedersen HK , Gudmundsdottir V, Nielsen HB et al; MetaHIT Consortium. Human gut microbes impact host serum metabolome and insulin sensitivity. Nature 2016;535:376–81. 27409811 10.1038/nature18646

[79] Scher JU , Sczesnak A, Longman RS et al Expansion of intestinal Prevotella copri correlates with enhanced susceptibility to arthritis. Elife 2013;2:e01202. 24192039 10.7554/eLife.01202 PMC3816614

[80] Moreno J. Prevotella copri and the microbial pathogenesis of rheumatoid arthritis. Reumatol Clin 2015;11:61–3. 25555460 10.1016/j.reuma.2014.11.001

[81] The Lancet Gastroenterology Hepatology. Probiotics: elixir or empty promise? Lancet Gastroenterol Hepatol 2019;4:81. 30647011 10.1016/S2468-1253(18)30415-1

[82] Spencer CN , McQuade JL, Gopalakrishnan V et al Dietary fiber and probiotics influence the gut microbiome and melanoma immunotherapy response. Science 2021;374:1632–40. 34941392 10.1126/science.aaz7015 PMC8970537

[83] Marchesi JR , Adams DH, Fava F et al The gut microbiota and host health: a new clinical frontier. Gut 2016;65:330–9. 26338727 10.1136/gutjnl-2015-309990 PMC4752653

[84] Jung TH , Park JH, Jeon WM et al Butyrate modulates bacterial adherence on LS174T human colorectal cells by stimulating mucin secretion and MAPK signaling pathway. Nutr Res Pract 2015;9:343–9. 26244071 10.4162/nrp.2015.9.4.343 PMC4523476

[85] Peng L , Li ZR, Green RS et al Butyrate enhances the intestinal barrier by facilitating tight junction assembly via activation of AMP-activated protein kinase in Caco-2 cell monolayers. J Nutr 2009;139:1619–25. 19625695 10.3945/jn.109.104638 PMC2728689

[86] Wu W , Sun M, Chen F et al Microbiota metabolite short-chain fatty acid acetate promotes intestinal IgA response to microbiota which is mediated by GPR43. Mucosal Immunol 2017;10:946–56. 27966553 10.1038/mi.2016.114 PMC5471141

[87] Liu P , Wang Y, Yang G et al The role of short-chain fatty acids in intestinal barrier function, inflammation, oxidative stress, and colonic carcinogenesis. Pharmacol Res 2021;165:105420. 33434620 10.1016/j.phrs.2021.105420

[88] Schauber J , Svanholm C, Termén S et al Expression of the cathelicidin LL-37 is modulated by short chain fatty acids in colonocytes: relevance of signalling pathways. Gut 2003;52:735–41. 12692061 10.1136/gut.52.5.735 PMC1773650

[89] Zhao Y , Chen F, Wu W et al GPR43 mediates microbiota metabolite SCFA regulation of antimicrobial peptide expression in intestinal epithelial cells via activation of mTOR and STAT3. Mucosal Immunol 2018;11:752–62. 29411774 10.1038/mi.2017.118 PMC5976519

[90] Smith PM , Howitt MR, Panikov N et al The microbial metabolites, short-chain fatty acids, regulate colonic Treg cell homeostasis. Science 2013;341:569–73. 23828891 10.1126/science.1241165 PMC3807819

[91] Furusawa Y , Obata Y, Fukuda S et al Commensal microbe-derived butyrate induces the differentiation of colonic regulatory T cells. Nature 2013;504:446–50. 24226770 10.1038/nature12721

[92] Arpaia N , Campbell C, Fan X et al Metabolites produced by commensal bacteria promote peripheral regulatory T-cell generation. Nature 2013;504:451–5. 24226773 10.1038/nature12726 PMC3869884

[93] Russell WR , Gratz SW, Duncan SH et al High-protein, reduced-carbohydrate weight-loss diets promote metabolite profiles likely to be detrimental to colonic health. Am J Clin Nutr 2011;93:1062–72. 21389180 10.3945/ajcn.110.002188

[94] Newgard CB , An J, Bain JR et al A branched-chain amino acid-related metabolic signature that differentiates obese and lean humans and contributes to insulin resistance. Cell Metab 2009;9:311–26. 19356713 10.1016/j.cmet.2009.02.002 PMC3640280

[95] Choi BS-Y , Daniel N, Houde VP et al Feeding diversified protein sources exacerbates hepatic insulin resistance via increased gut microbial branched-chain fatty acids and mTORC1 signaling in obese mice. Nat Commun 2021;12:3377. 34099716 10.1038/s41467-021-23782-w PMC8184893

[96] Chassaing B , Koren O, Goodrich JK et al Dietary emulsifiers impact the mouse gut microbiota promoting colitis and metabolic syndrome. Nature 2015;519:92–6. 25731162 10.1038/nature14232 PMC4910713

[97] Chassaing B , Compher C, Bonhomme B et al Randomized controlled-feeding study of dietary emulsifier carboxymethylcellulose reveals detrimental impacts on the gut microbiota and metabolome. Gastroenterology 2021;**162**:743–56. 10.1053/j.gastro.2021.11.006 PMC9639366 34774538

[98] Hancock RE , Haney EF, Gill EE. The immunology of host defence peptides: beyond antimicrobial activity. Nat Rev Immunol 2016;16:321–34. 27087664 10.1038/nri.2016.29

[99] Selsted ME , Ouellette AJ. Mammalian defensins in the antimicrobial immune response. Nat Immunol 2005;6:551–7. 15908936 10.1038/ni1206

[100] White SH , Wimley WC, Selsted ME. Structure, function, and membrane integration of defensins. Curr Opin Struct Biol 1995;5:521–7. 8528769 10.1016/0959-440x(95)80038-7

[101] Bevins CL , Salzman NH. Paneth cells, antimicrobial peptides and maintenance of intestinal homeostasis. Nat Rev Microbiol 2011;9:356–68. 21423246 10.1038/nrmicro2546

[102] Antoni L , Nuding S, Weller D et al Human colonic mucus is a reservoir for antimicrobial peptides. J Crohns Colitis 2013;7:e652–64. 23787054 10.1016/j.crohns.2013.05.006

[103] Mukherjee S , Zheng H, Derebe MG et al Antibacterial membrane attack by a pore-forming intestinal C-type lectin. Nature 2014;505:103–7. 24256734 10.1038/nature12729 PMC4160023

[104] Wohlkönig A , Huet J, Looze Y et al Structural relationships in the lysozyme superfamily: significant evidence for glycoside hydrolase signature motifs. PLoS One 2010;5:e15388. 21085702 10.1371/journal.pone.0015388 PMC2976769

[105] Primo ED , Otero LH, Ruiz F et al The disruptive effect of lysozyme on the bacterial cell wall explored by an in-silico structural outlook. Biochem Mol Biol Educ 2018;46:83–90. 29131507 10.1002/bmb.21092

[106] Chu H , Pazgier M, Jung G et al Human alpha-defensin 6 promotes mucosal innate immunity through self-assembled peptide nanonets. Science 2012;337:477–81. 22722251 10.1126/science.1218831 PMC4332406

[107] Ebenhan T , Gheysens O, Kruger HG et al Antimicrobial peptides: their role as infection-selective tracers for molecular imaging. Biomed Res Int 2014;2014:867381. 25243191 10.1155/2014/867381 PMC4163393

[108] Yeaman MR , Yount NY. Mechanisms of antimicrobial peptide action and resistance. Pharmacol Rev 2003;55:27–55. 12615953 10.1124/pr.55.1.2

[109] Salzman NH , Bevins CL. Dysbiosis-a consequence of Paneth cell dysfunction. Semin Immunol 2013;25:334–41. 24239045 10.1016/j.smim.2013.09.006

[110] Salzman NH , Hung K, Haribhai D et al Enteric defensins are essential regulators of intestinal microbial ecology. Nat Immunol 2010;11:76–82. 19855381 10.1038/ni.1825 PMC2795796

[111] Petnicki-Ocwieja T , Hrncir T, Liu Y-J et al Nod2 is required for the regulation of commensal microbiota in the intestine. Proc Natl Acad Sci USA 2009;106:15813–8. 19805227 10.1073/pnas.0907722106 PMC2747201

[112] Liu T-C , Kern JT, Jain U et al Western diet induces Paneth cell defects through microbiome alterations and farnesoid X receptor and type I interferon activation. Cell Host Microbe 2021;29:988–1001.e6. 34010595 10.1016/j.chom.2021.04.004 PMC8192497

[113] Vaishnava S , Behrendt CL, Ismail AS et al Paneth cells directly sense gut commensals and maintain homeostasis at the intestinal host-microbial interface. Proc Natl Acad Sci USA 2008;105:20858–63. 19075245 10.1073/pnas.0808723105 PMC2603261

[114] Larsen IS , Jensen BAH, Bonazzi E et al Fungal lysozyme leverages the gut microbiota to curb DSS-induced colitis. Gut Microbes 2021;13:1988836. 34693864 10.1080/19490976.2021.1988836 PMC8547870

[115] Zigdon M , Bel S. Lysozyme: a double-edged sword in the intestine. Trends Immunol 2020;41:1054–6. 33158739 10.1016/j.it.2020.10.010

[116] Yu S , Balasubramanian I, Laubitz D et al Paneth cell-derived lysozyme defines the composition of mucolytic microbiota and the inflammatory tone of the intestine. Immunity 2020;53:398–416.e8. 32814028 10.1016/j.immuni.2020.07.010 PMC7461615

[117] Mookherjee N , Anderson MA, Haagsman HP et al Antimicrobial host defence peptides: functions and clinical potential. Nat Rev Drug Discov 2020;19:311–32. 32107480 10.1038/s41573-019-0058-8

[118] Andersson DI , Hughes D, Kubicek-Sutherland JZ. Mechanisms and consequences of bacterial resistance to antimicrobial peptides. Drug Resist Updat 2016;26:43–57. 27180309 10.1016/j.drup.2016.04.002

[119] Wang G , Li X, Wang Z. APD3: the antimicrobial peptide database as a tool for research and education. Nucleic Acids Res 2016;44:D1087–93. 26602694 10.1093/nar/gkv1278 PMC4702905

[120] Ehmann D , Wendler J, Koeninger L et al Paneth cell α-defensins HD-5 and HD-6 display differential degradation into active antimicrobial fragments. Proc Natl Acad Sci USA 2019;116:3746–51. 30808760 10.1073/pnas.1817376116 PMC6397583

[121] Ehmann D , Koeninger L, Wendler J et al Fragmentation of human neutrophil alpha-defensin 4 to combat multidrug resistant bacteria. Front Microbiol 2020;11:1147. 32582092 10.3389/fmicb.2020.01147 PMC7286198

[122] Wendler J , Schroeder BO, Ehmann D et al Proteolytic degradation of reduced human beta defensin 1 generates a novel antibiotic octapeptide. Sci Rep 2019;9:3640. 30842543 10.1038/s41598-019-40216-2 PMC6403363

[123] Zhang Y , Cougnon FB, Wanniarachchi YA et al Reduction of human defensin 5 affords a high-affinity zinc-chelating peptide. ACS Chem Biol 2013;8:1907–11. 23841778 10.1021/cb400340k PMC3783636

[124] Jaeger SU , Schroeder BO, Meyer-Hoffert U et al Cell-mediated reduction of human beta-defensin 1: a major role for mucosal thioredoxin. Mucosal Immunol 2013;6:1179–90. 23571504 10.1038/mi.2013.17 PMC3806438

[125] Schroeder BO , Wu Z, Nuding S et al Reduction of disulphide bonds unmasks potent antimicrobial activity of human beta-defensin 1. Nature 469:419–23. 2011; 21248850 10.1038/nature09674

[126] Schroeder BO , Stange EF, Wehkamp J. Waking the wimp: redox-modulation activates human beta-defensin 1. Gut Microbes 2011;2:262–6. 21983064 10.4161/gmic.2.4.17692

[127] Schmidtchen A , Frick IM, Andersson E et al Proteinases of common pathogenic bacteria degrade and inactivate the antibacterial peptide LL-37. Mol Microbiol 2002;46:157–68. 12366839 10.1046/j.1365-2958.2002.03146.x

[128] Phan TK , Bevins CL, Hulett MD. Editorial: Advances in the immunology of host defense peptide: mechanisms and applications of antimicrobial functions and beyond. Front Immunol 2021;12:637641. 33717187 10.3389/fimmu.2021.637641 PMC7947235

[129] Koeninger L , Armbruster NS, Brinch KS et al Human β-defensin 2 mediated immune modulation as treatment for experimental colitis. Front Immunol 2020;11:93. 32076420 10.3389/fimmu.2020.00093 PMC7006816

[130] Pinkerton JW , Kim RY, Koeninger L et al Human β‐defensin‐2 suppresses key features of asthma in murine models of allergic airways disease. Clin Exp Allergy 2021;51:120–31. 33098152 10.1111/cea.13766

[131] Borchers NS , Santos-Valente E, Toncheva AA et al Human β-defensin 2 mutations are associated with asthma and atopy in children and its application prevents atopic asthma in a mouse model. Front Immunol 2021;12:636061. 33717182 10.3389/fimmu.2021.636061 PMC7946850

[132] Ghosh SK , Weinberg A. Ramping up antimicrobial peptides against severe acute respiratory syndrome coronavirus-2. Front Mol Biosci 2021;8:620806. 34235176 10.3389/fmolb.2021.620806 PMC8255374

[133] Liang W , Enée E, Andre-Vallee C et al Intestinal cathelicidin antimicrobial peptide shapes a protective neonatal gut microbiota against pancreatic autoimmunity. Gastroenterology 2021, 10.1053/j.gastro.2021.12.272. 10.1053/j.gastro.2021.12.272 34973295

[134] Röhrl J , Yang D, Oppenheim JJ et al Human β-defensin 2 and 3 and their mouse orthologs induce chemotaxis through interaction with CCR2. J Immunol 2010;184:6688–94. 20483750 10.4049/jimmunol.0903984 PMC6309988

[135] Röhrl J , Yang D, Oppenheim JJ et al Specific binding and chemotactic activity of mBD4 and its functional orthologue hBD2 to CCR6-expressing cells. J Biol Chem 2010;285:7028–34. 20068036 10.1074/jbc.M109.091090 PMC2844152

[136] Zhan Y , Wang N, Vasanthakumar A et al CCR2 enhances CD25 expression by FoxP3+ regulatory T cells and regulates their abundance independently of chemotaxis and CCR2+ myeloid cells. Cell Mol Immunol 2020;17:123–32. 30538272 10.1038/s41423-018-0187-8 PMC7000403

[137] Jørgensen AS , Brandum EP, Mikkelsen JM et al The C-terminal peptide of CCL21 drastically augments CCL21 activity through the dendritic cell lymph node homing receptor CCR7 by interaction with the receptor N-terminus. Cell Mol Life Sci 2021;78:6963–78. 34586443 10.1007/s00018-021-03930-7 PMC8558179

[138] Loddo I , Romano C. Inflammatory bowel disease: genetics, epigenetics, and pathogenesis. Front Immunol 2015;6:551. 26579126 10.3389/fimmu.2015.00551 PMC4629465

[139] Caruso R , Mathes T, Martens EC et al A specific gene-microbe interaction drives the development of Crohn’s disease-like colitis in mice. Sci Immunol 2019;4:eaaw4341. 10.1126/sciimmunol.aaw4341 PMC8882361 31004013

[140] Hugot JP , Chamaillard M, Zouali H et al Association of NOD2 leucine-rich repeat variants with susceptibility to Crohn’s disease. Nature 2001;411:599–603. 11385576 10.1038/35079107

[141] Ogura Y , Bonen DK, Inohara N et al A frameshift mutation in NOD2 associated with susceptibility to Crohn’s disease. Nature 2001;411:603–6. 11385577 10.1038/35079114

[142] Kobayashi KS , Chamaillard M, Ogura Y et al Nod2-dependent regulation of innate and adaptive immunity in the intestinal tract. Science 2005;307:731–4. 15692051 10.1126/science.1104911

[143] Ogura Y , Lala S, Xin W et al Expression of NOD2 in Paneth cells: a possible link to Crohn’s ileitis. Gut 2003;52:1591–7. 14570728 10.1136/gut.52.11.1591 PMC1773866

[144] Wehkamp J , Stange EF. An update review on the Paneth cell as key to ileal Crohn’s. Front Immunol 2020;11:646. 32351509 10.3389/fimmu.2020.00646 PMC7174711

[145] Cadwell K , Liu JY, Brown SL et al A key role for autophagy and the autophagy gene Atg16l1 in mouse and human intestinal Paneth cells. Nature 2008;456:259–63. 18849966 10.1038/nature07416 PMC2695978

[146] Aden K , Tran F, Ito G et al ATG16L1 orchestrates interleukin-22 signaling in the intestinal epithelium via cGAS-STING. J Exp Med 2018;215:2868–86. 30254094 10.1084/jem.20171029 PMC6219748

[147] Britton GJ , Contijoch EJ, Mogno I et al Microbiotas from humans with inflammatory bowel disease alter the balance of Gut Th17 and RORγt+ regulatory T cells and exacerbate colitis in Mice. Immunity 2019;50:212–24.e4. 30650377 10.1016/j.immuni.2018.12.015 PMC6512335

[148] Zeng MY , Cisalpino D, Varadarajan S et al Gut microbiota-induced immunoglobulin G controls systemic infection by symbiotic bacteria and pathogens. Immunity 2016;44:647–58. 26944199 10.1016/j.immuni.2016.02.006 PMC4794373

[149] Peyrin-Biroulet L , Gonzalez F, Dubuquoy L et al Mesenteric fat as a source of C reactive protein and as a target for bacterial translocation in Crohn’s disease. Gut 2012;61:78–85. 21940721 10.1136/gutjnl-2011-300370 PMC3230831

[150] Atreya R , Siegmund B. Location is important: differentiation between ileal and colonic Crohn’s disease. Nat Rev Gastroenterol Hepatol 2021;18:544–58. 33712743 10.1038/s41575-021-00424-6

[151] Ha CWY , Martin A, Sepich-Poore GD et al Translocation of viable gut microbiota to mesenteric adipose drives formation of creeping fat in humans. Cell 2020;183:666–83.e17. 32991841 10.1016/j.cell.2020.09.009 PMC7521382

[152] He Z , Wu J, Gong J et al Microbiota in mesenteric adipose tissue from Crohn’s disease promote colitis in mice. Microbiome 2021;9:228. 34814945 10.1186/s40168-021-01178-8 PMC8609859

[153] Massier L , Chakaroun R, Tabei S et al Adipose tissue derived bacteria are associated with inflammation in obesity and type 2 diabetes. Gut 2020;69:1796–806. 32317332 10.1136/gutjnl-2019-320118

[154] Anhê FF , Jensen BAH, Varin TV et al Type 2 diabetes influences bacterial tissue compartmentalisation in human obesity. Nature Metabol 2020;2:233–42. 10.1038/s42255-020-0178-9 32694777

[155] Suppli MP , Bagger JI, Lelouvier B et al Hepatic microbiome in healthy lean and obese humans. JHEP Rep 2021;3:100299. 34169247 10.1016/j.jhepr.2021.100299 PMC8207208

[156] Sookoian S , Salatino A, Castaño GO et al Intrahepatic bacterial metataxonomic signature in non-alcoholic fatty liver disease. Gut 2020;69:1483–91. 31900291 10.1136/gutjnl-2019-318811

[157] Jensen BAH , Marette A. Microbial translocation in type 2 diabetes: when bacterial invaders overcome host defence in human obesity. Gut 2020;69:1724–6. 32518079 10.1136/gutjnl-2020-321288

[158] Massier L , Blüher M, Kovacs P et al Impaired intestinal barrier and tissue bacteria: pathomechanisms for metabolic diseases. Front Endocrinol (Lausanne) 2021;12:616506. 33767669 10.3389/fendo.2021.616506 PMC7985551

[159] Viennois E , Chassaing B. First victim, later aggressor: how the intestinal microbiota drives the pro-inflammatory effects of dietary emulsifiers? Gut Microbes 2018;9:289–91. 10.1080/19490976.2017.1421885 PMC6219590 29437527

[160] Larsen IS , Fritzen AM, Carl CS et al Human Paneth cell alpha-defensin-5 treatment reverses dyslipidemia and improves glucoregulatory capacity in diet-induced obese mice. Am J Physiol Endocrinol Metab 2019;317:E42–52. 30860877 10.1152/ajpendo.00019.2019

[161] Thaiss CA , Levy M, Grosheva I et al Hyperglycemia drives intestinal barrier dysfunction and risk for enteric infection. Science 2018;359:1376–83. 29519916 10.1126/science.aar3318

[162] Chakaroun RM , Massier L, Kovacs P. Gut microbiome, intestinal permeability, and tissue bacteria in metabolic disease: perpetrators or bystanders? Nutrients 2020;12:1082. 10.3390/nu12041082 PMC7230435 32295104

[163] Luo Z , Ji Y, Gao H et al CRIg+ macrophages prevent gut microbial DNA-containing extracellular vesicle-induced tissue inflammation and insulin resistance. Gastroenterology 2021;160:863–74. 33152356 10.1053/j.gastro.2020.10.042 PMC7878308

[164] Boulenouar S , Michelet X, Duquette D et al Adipose type one innate lymphoid cells regulate macrophage homeostasis through targeted cytotoxicity. Immunity 2017;46:273–86. 28228283 10.1016/j.immuni.2017.01.008

[165] Cani PD , Amar J, Iglesias MA et al Metabolic endotoxemia initiates obesity and insulin resistance. Diabetes 2007;56:1761–72. 17456850 10.2337/db06-1491

[166] Lassenius MI , Pietiläinen KH, Kaartinen K et al; on behalf of the FinnDiane Study Group. Bacterial endotoxin activity in human serum is associated with dyslipidemia, insulin resistance, obesity, and chronic inflammation. Diabetes Care 2011;34:1809–15. 21636801 10.2337/dc10-2197 PMC3142060

[167] Harte AL , Varma MC, Tripathi G et al High fat intake leads to acute postprandial exposure to circulating endotoxin in type 2 diabetic subjects. Diabetes Care 2012;35:375–82. 22210577 10.2337/dc11-1593 PMC3263907

[168] Pearson CR , Tindall SN, Herman R et al Acetylation of surface carbohydrates in bacterial pathogens requires coordinated action of a two-domain membrane-bound acyltransferase. mBio 2020;11:e01364–20. 32843546 10.1128/mBio.01364-20 PMC7448272

[169] Anhê FF , Barra NG, Cavallari JF et al Metabolic endotoxemia is dictated by the type of lipopolysaccharide. Cell Rep 2021;36:109691. 34525353 10.1016/j.celrep.2021.109691

[170] Ohnmacht C , Park J-H, Cording S et al Mucosal immunology: the microbiota regulates type 2 immunity through RORγt+ T cells. Science 2015;349:989–93. 26160380 10.1126/science.aac4263

[171] Yang B-H , Hagemann S, Mamareli P et al Foxp3+ T cells expressing RORγt represent a stable regulatory T-cell effector lineage with enhanced suppressive capacity during intestinal inflammation. Mucosal Immunol 2016;9:444–57. 26307665 10.1038/mi.2015.74

[172] Sefik E , Geva-Zatorsky N, Oh S et al Mucosal immunology: individual intestinal symbionts induce a distinct population of RORγ+ regulatory T cells. Science 2015;349:993–7. 26272906 10.1126/science.aaa9420 PMC4700932

[173] Song X , Sun X, Oh SF et al Microbial bile acid metabolites modulate gut RORγ+regulatory T cell homeostasis. Nature 2020;577:410–5. 31875848 10.1038/s41586-019-1865-0 PMC7274525

[174] Wang S , Charbonnier L-M, Noval Rivas M et al MyD88 adaptor-dependent microbial sensing by regulatory T cells promotes mucosal tolerance and enforces commensalism. Immunity 2015;43:289–303. 26231118 10.1016/j.immuni.2015.06.014 PMC4545404

[175] Ohnmacht C , Park JH, Cording S et al The microbiota regulates type 2 immunity through RORγt+ T cells. Science 2015;349:989–93. 26160380 10.1126/science.aac4263

[176] Agace WW , McCoy KD. Regionalized development and maintenance of the intestinal adaptive immune landscape. Immunity 2017;46:532–48. 28423335 10.1016/j.immuni.2017.04.004

[177] Tanoue T , Atarashi K, Honda K. Development and maintenance of intestinal regulatory T cells. Nat Rev Immunol 2016;16:295–309. 27087661 10.1038/nri.2016.36

[178] Esterházy D , Canesso MCC, Mesin L et al Compartmentalized gut lymph node drainage dictates adaptive immune responses. Nature 2019;569:126–30. 30988509 10.1038/s41586-019-1125-3 PMC6587593

[179] Wang K , Liao M, Zhou N et al Parabacteroides distasonis alleviates obesity and metabolic dysfunctions via production of succinate and secondary bile acids. Cell Rep 2019;26:222–35.e5. 30605678 10.1016/j.celrep.2018.12.028

[180] Kverka M , Zakostelska Z, Klimesova K et al Oral administration of Parabacteroides distasonis antigens attenuates experimental murine colitis through modulation of immunity and microbiota composition. Clin Exp Immunol 2011;163:250–9. 21087444 10.1111/j.1365-2249.2010.04286.x PMC3043316

[181] Kleiveland CR , Hult LTO, Spetalen S et al The Noncommensal Bacterium Methylococcus capsulatus (Bath) ameliorates dextran sulfate (sodium salt)-induced ulcerative colitis by influencing mechanisms essential for maintenance of the colonic barrier function. Appl Environ Microbiol 2013;79:48–56. 23064342 10.1128/AEM.02464-12 PMC3536074

[182] Rowan F , Docherty NG, Murphy M et al Desulfovibrio bacterial species are increased in ulcerative colitis. Dis Colon Rectum 2010;53:1530–6. 20940602 10.1007/DCR.0b013e3181f1e620

[183] Petersen C , Bell R, Klag KA et al T cell-mediated regulation of the microbiota protects against obesity. Science 2019;365:eaat9351. 31346040 10.1126/science.aat9351 PMC7294966

[184] Heidelberg JF , Seshadri R, Haveman SA et al The genome sequence of the anaerobic, sulfate-reducing bacterium Desulfovibrio vulgaris Hildenborough. Nat Biotechnol 2004;22:554–9. 15077118 10.1038/nbt959

[185] Lennon G , Balfe Á, Bambury N et al Correlations between colonic crypt mucin chemotype, inflammatory grade and Desulfovibrio species in ulcerative colitis. Colorectal Dis 2014;16:O161–9. 24345279 10.1111/codi.12503

[186] Dordević D , Jančíková S, Vítězová M et al Hydrogen sulfide toxicity in the gut environment: meta-analysis of sulfate-reducing and lactic acid bacteria in inflammatory processes. J Adv Res 2021;27:55–69. 33318866 10.1016/j.jare.2020.03.003 PMC7728594

